# The PIWI protein Aubergine recruits eIF3 to activate translation in the germ plasm

**DOI:** 10.1038/s41422-020-0294-9

**Published:** 2020-03-04

**Authors:** Anne Ramat, Maria-Rosa Garcia-Silva, Camille Jahan, Rima Naït-Saïdi, Jérémy Dufourt, Céline Garret, Aymeric Chartier, Julie Cremaschi, Vipul Patel, Mathilde Decourcelle, Amandine Bastide, François Juge, Martine Simonelig

**Affiliations:** 1 mRNA Regulation and Development, Institute of Human Genetics, UMR9002 CNRS-Univ Montpellier, 141 rue de la Cardonille, 34396 Montpellier Cedex 5, France; 2 BioCampus Montpellier, CNRS, INSERM, Univ Montpellier, Montpellier, France; 3 IGF, Univ Montpellier, CNRS, INSERM, Montpellier, France; 4 Institut de Génétique Moléculaire de Montpellier, Univ Montpellier, CNRS, Montpellier, France; 5 Present Address: Institut de Génétique Moléculaire de Montpellier, Univ Montpellier, CNRS, Montpellier, France

**Keywords:** Piwi RNAs, Developmental biology

## Abstract

Piwi-interacting RNAs (piRNAs) and PIWI proteins are essential in germ cells to repress transposons and regulate mRNAs. In *Drosophila*, piRNAs bound to the PIWI protein Aubergine (Aub) are transferred maternally to the embryo and regulate maternal mRNA stability through two opposite roles. They target mRNAs by incomplete base pairing, leading to their destabilization in the soma and stabilization in the germ plasm. Here, we report a function of Aub in translation. Aub is required for translational activation of *nanos* mRNA, a key determinant of the germ plasm. Aub physically interacts with the poly(A)-binding protein (PABP) and the translation initiation factor eIF3. Polysome gradient profiling reveals the role of Aub at the initiation step of translation. In the germ plasm, PABP and eIF3d assemble in foci that surround Aub-containing germ granules, and Aub acts with eIF3d to promote *nanos* translation. These results identify translational activation as a new mode of mRNA regulation by Aub, highlighting the versatility of PIWI proteins in mRNA regulation.

## Introduction

Translational control is a widespread mechanism to regulate gene expression in many biological contexts. This regulation has an essential role during early embryogenesis, before transcription of the zygotic genome has actually started. In *Drosophila*, embryonic patterning depends on the translational control of a small number of maternal mRNAs.^1^ Among them, *nanos* (*nos*) mRNA encodes a key posterior determinant required for abdominal segmentation and development of the germline.^2^ *nos* mRNA is present in the whole embryo, but a small proportion accumulates at the posterior pole in the germ plasm, a specialized cytoplasm in which the germline develops.^3,4^ Localization and translational control of *nos* mRNA are linked, such that the pool of *nos* mRNA present in the bulk of the embryo is translationally repressed, whereas the pool of *nos* mRNA localized in the germ plasm is translationally activated to produce a Nos protein gradient from the posterior pole.^3,5,6^ Both repression of *nos* mRNA translation in the bulk of the embryo and activation in the germ plasm are required for embryonic development.

The coupling between mRNA localization and translational control depends in part on the implication of the same factors in both processes. The Smaug (Smg) RNA binding protein specifically recognizes *nos* mRNA through binding to two Smaug recognition elements (SRE) in its 3′UTR.^7,8^ Smg is both a translational repressor of *nos*, and a localization factor through its role in mRNA deadenylation and decay in the bulk of the embryo, by recruitment of the CCR4-NOT deadenylation complex.^7–9^ Smg directly interacts with the Oskar (Osk) protein that is specifically synthesized at the posterior pole of oocytes and embryos and drives germ plasm assembly.^7,10^ Smg interaction with Osk prevents Smg binding to *nos* mRNA, thus contributing to relieving both Smg-dependent translational repression and mRNA decay in the germ plasm.^7,9,11^ Osk is therefore a key player in the switch of *nos* and other germ cell mRNA regulation between soma and germ plasm of the embryo.

More recently, we have demonstrated the role of Aubergine (Aub) in the localization of germ cell mRNAs to the germ plasm.^12,13^ Aub is one of the three PIWI proteins in *Drosophila*. PIWI proteins belong to a specific clade of Argonaute proteins that bind 23–30 nucleotides (nt)-long small RNAs referred to as Piwi-interacting RNAs (piRNAs).^14,15^ piRNAs and PIWI proteins have an established role in the repression of transposable elements in the germline of animals. piRNAs target transposable element mRNAs through complementarity and guide interaction with PIWI proteins that, in turn, cleave targeted mRNAs through their endonucleolytic activity. In addition to this role, piRNAs have a conserved function in the regulation of cellular mRNAs in various biological contexts.^16^ In the *Drosophila* embryo, Aub loaded with piRNAs produced in the female germline is present both at low levels in the bulk of the embryo and at higher levels in the germ plasm.^17,18^ Aub binds maternal germ cell mRNAs through incomplete base pairing with piRNAs.^12,18,19^ Aub binding to these mRNAs induces their decay in the bulk of the embryo, either by direct cleavage or recruitment together with Smg of the CCR4-NOT deadenylation complex.^12,18^ In contrast, in the germ plasm Aub recruits Wispy, the germline-specific cytoplasmic poly(A) polymerase, leading to poly(A) tail elongation and stabilization of Aub-bound mRNAs.^13^ Thus, Aub and piRNAs play a central role in the localization of germ cell mRNAs through two opposite functions in mRNA stability: mRNA destabilization in the bulk of the embryo and stabilization in the germ plasm. The role of piRNAs and PIWI proteins in cellular mRNA regulation in other contexts, including mouse spermiogenesis and sex determination in *Bombyx*, also depends on their function in the regulation of mRNA stability.^20–24^

Here, we describe translational activation as a new mechanism of mRNA regulation by piRNAs and PIWI proteins. Using ectopic expression of Osk in the whole embryo to mimic the germ plasm, we show that Aub and piRNAs are required for *nos* mRNA translation. Mass spectrometry analysis of Aub interactors in early embryos identifies several components of the translation machinery, including translation initiation factors. We find that Aub physically interacts with the poly(A)-binding protein (PABP) and several subunits of the translation initiation complex eIF3. Furthermore, PABP and eIF3d accumulate in foci that assemble around and partially overlap with Aub-containing germ granules in the germ plasm. Polysome gradient analysis indicates that Aub activates translation at the initiation step. Finally, functional experiments involving the concomitant decrease of Aub and eIF3d show that both proteins act together in *nos* mRNA translation in the germ plasm. These results identify translational activation as a new level of mRNA regulation by PIWI proteins. Moreover, they expand the role of the general eIF3 translation initiation complex in translation regulatory mechanisms required for developmental processes.

## Results

### Aub is required for *nos* mRNA translation

**Fig. 1:**
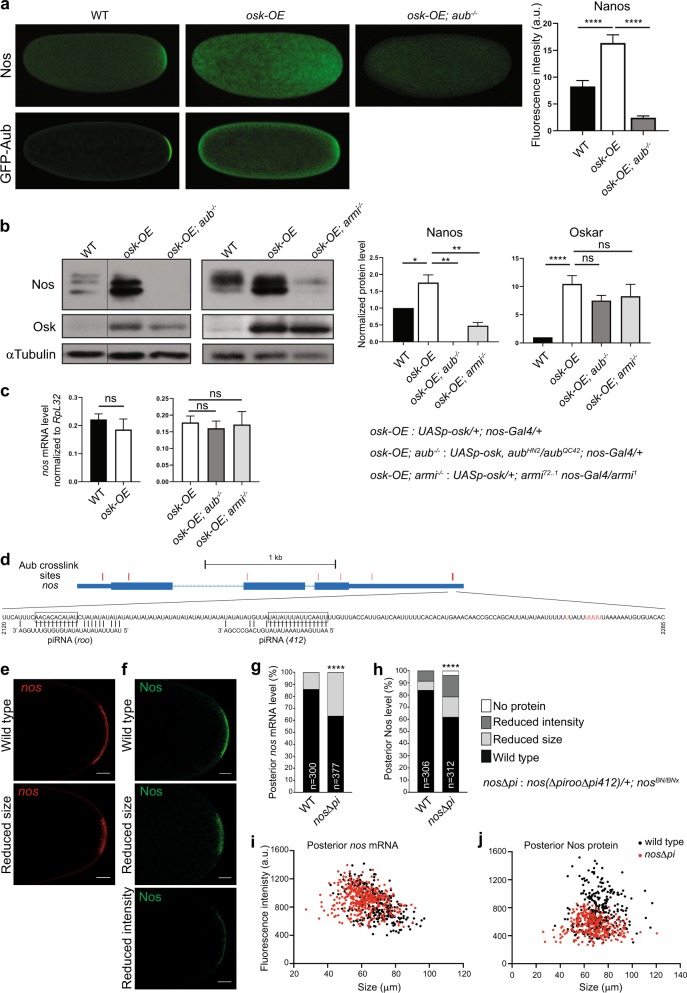
Aub and Armi are required for *nos* mRNA translation. **a** Immunostaining of WT, *osk-OE* and *osk-OE; aub*^*−/−*^ embryos with anti-Nos antibody (top panels). The genotypes are indicated. Fluorescence quantification was performed using the ImageJ software with 5–6 embryos per genotype. Error bars represent SEM. ****P < 0.0001 using the unpaired Student’s *t*-test. Immunostaining of *UASp-GFP-Aub nos-Gal4* and *UASp-osk/+; UASp-GFP-Aub nos-Gal4/+* embryos with anti-GFP antibody, showing the distribution of Aub protein (bottom panels). Posterior is to the right. **b** Western blots of WT, *osk-OE*, *osk-OE; aub*^*−/−*^ and *osk-OE*; *armi*^*−/−*^ embryos revealed with anti-Nos, anti-Osk and anti-α-Tubulin antibodies. α-Tubulin was used as a loading control. Quantification was performed using the ImageJ software with 3–6 biological replicates. Error bars represent SEM. ****P < 0.0001, **P < 0.01, *P < 0.05, ns not significant, using the unpaired Student’s *t*-test. **c** Quantification of *nos* mRNA using RT-qPCR in WT, *osk-OE*, *osk-OE; aub*^*−/−*^ and *osk-OE; armi*^*−/−*^ embryos. mRNA levels were normalized with *RpL32* mRNA. Quantification of 4–8 biological replicates. Error bars represent SEM. ns not significant, using the unpaired Student’s *t*-test. **d** Schematic representation of *nos* mRNA and 3′UTR targeting with piRNAs. Thin boxes are 5′UTR and 3′UTR, lines are introns and thick boxes are exons. Clusters of Aub crosslink sites are indicated in red.^12^ The sequence of the region with the strongest crosslink sites and base pairing with representative *roo* and *412* piRNAs are shown. The deletions overlapping with the piRNA target sites in the *nos(ΔpirooΔpi412)* transgene are boxed.^18^ Aub crosslinked nt are in red. **e**, **f** *nos* smFISH (**e**) and immunostaining with anti**-**Nos antibody (**f**) of wild-type and *nos(ΔpirooΔpi412)/+; nos*^*BN/BNx*^ embryos. Posterior of embryos with the three types of staining: wild type, reduced size or reduced intensity, are shown. Scale bars, 20 μm. **g**–**j** Quantification of posterior staining shown in **e** and **f** using the ImageJ software. For each genotype, the percentage of embryos with each staining category was recorded for *nos* mRNA (**g**) and Nos protein (**h**). ****P < 0.0001 using the χ^2^ test. Scatter plots of size and fluorescence intensity of posterior staining for each embryo, for *nos* mRNA (**i**) and Nos protein (**j**). Two-way ANOVA showed significant difference (*P* < 0.001) in fluorescence intensity of posterior staining for *nos* mRNA and Nos protein between genotypes.

Only a low amount (4%) of *nos* mRNA is localized to the germ plasm and actually translated.^3,4^ *nos* mRNA stabilization and translation in the germ plasm depend on the presence of Osk. Therefore, we ectopically expressed Osk in the whole embryo using *UASp-osk*^25^ and the germline-specific driver *nos-Gal4*, to increase translated *nos* mRNA levels and address the mechanisms of translational activation. Osk overexpression (*osk-OE*) in embryos from *UASp-osk/+; nos-Gal4/+* females led to increased and ectopic Nos protein synthesis in whole embryo (Fig. 1a). Quantification of Nos protein levels in *osk-OE* embryos, either following Nos visualization using immunostaining or western blot, revealed a 2-fold increase compared to wild-type (WT) embryos (Fig. 1a, b). In contrast, *nos* mRNA levels quantified using RT-qPCR were similar in *osk-OE* and WT embryos (Fig. 1c). This is consistent with the presence of high amounts of *nos* mRNA in the bulk of embryos, and *nos* spatial regulation depending mostly on translational control at these stages (0–2 h embryos). Therefore, Osk overexpression in 0–2 h embryos led to ectopic translational activation of *nos* mRNA without changes in *nos* mRNA levels.

Aub protein is present at low levels in the bulk of WT embryos and highly accumulates in the germ plasm.^18^ Ectopic expression of Osk led to an homogeneous redistribution of Aub in the embryo (Fig. 1a). Strikingly, the lack of Aub in *osk-OE* embryos resulted in the lack of Nos protein synthesis (Fig. 1a, b), although *nos* mRNA levels were not decreased (Fig. 1c). This result suggested that Aub was required for *nos* mRNA translational activation in the presence of Osk. Importantly, the level of Osk protein was not significantly affected by *aub* mutation, indicating that the lack of Nos protein did not result from the lack of Osk (Fig. 1b). Of note, the *UASp-osk* transgene almost exclusively overexpressed the long Osk isoform of the two isoforms, Short-Osk and Long-Osk (Supplementary information, Fig. S1a). Long-Osk can induce germ plasm assembly when overexpressed although less actively than Short-Osk.^26^ Long-Osk levels were poorly affected by *aub* mutations, making this *UASp-osk* transgene a useful tool to address direct *nos* mRNA regulation by Aub and piRNAs (Fig. 1b; Supplementary information, Fig. S1a).

We analyzed the role of Armitage (Armi), another component of the piRNA pathway with a prominent role in piRNA biogenesis,^27^ in *nos* mRNA translational activation. Nos protein levels were strongly reduced in *osk-OE; armi*^*−/−*^ embryos, compared to *osk-OE* embryos, although again, the levels of Osk protein were not significantly decreased (Fig. 1b). *nos* mRNA levels quantified using RT-qPCR remained unaffected by *armi* mutation (Fig. 1c), revealing a role of Armi in *nos* mRNA translational control. Armi does not localize to the germ plasm.^13^ Instead the defect in *nos* mRNA translational activation in *armi* mutant might depend on highly reduced piRNA levels in this mutant,^27^ suggesting that piRNAs were required in Aub binding to *nos* mRNA for its role in translational activation. This is consistent with Aub iCLIP assays showing that an Aub double point mutant in the PAZ domain, Aub^AA^ that is unable to load piRNAs, was also unable to bind mRNAs.^12^ To confirm the role of piRNAs in Aub-dependent translational activation of *nos*, we took advantage of the *nos(ΔpirooΔpi412)* transgene, in which two piRNA target sites (from *roo* and *412* transposable elements) in close proximity to a prominent Aub-binding site in *nos* 3′UTR have been deleted^12,18^ (Fig. 1d). We have shown before that deletion of these piRNA target sites affected *nos* mRNA localization to the germ plasm, without affecting its level.^13^ Using single molecule fluorescence *in situ* hybridization (smFISH), we confirmed the posterior localization defect of *nos* mRNA from this transgene: 36% of embryos showed a reduced domain of posterior localization (Fig. 1e, g, i). As previously reported, this defect was moderate because several Aub-binding sites remained unaffected in the *nos(ΔpirooΔpi412)* transgene (Fig. 1d).^13^ Recording *nos* mRNA translation in *nos(ΔpirooΔpi412)/+; nos*^*BN/BNx*^ embryos using immunostaining showed a similar percentage of embryos (38%) with reduced protein accumulation (Fig. 1f, h, j). However, protein synthesis appeared to be more affected than mRNA localization in these embryos since 3.8% of them did not produce any Nos protein, a defect (no localization) that did not occur with *nos* mRNA (Fig. 1e, g). In addition, when taking into account all embryos, immunofluorescence intensity was reduced in *nos(ΔpirooΔpi412)/+; nos*^*BN/BNx*^ embryos compared to WT, again a defect that did not occur with smFISH (Fig. 1i, j). Therefore, deletion of piRNA target sites in *nos* mRNA affected Nos protein synthesis, in addition to reducing mRNA localization.

These data are consistent with a direct role of Aub and piRNAs in *nos* mRNA translation through piRNA-guided binding of Aub to *nos*. In this hypothesis, a piRNA pathway component specifically involved in transposable element regulation should not interfere with *nos* mRNA translational control. We used Panoramix (Panx), a key factor in Piwi-dependent transcriptional silencing of transposable elements, which acts downstream of Piwi and has no function in piRNA biogenesis.^28,29^ *panx* mutants had no effect on Nos protein levels in *osk-OE* embryos, consistent with a role of Aub and piRNAs in *nos* mRNA translation, independent of their role in transposable element regulation (Supplementary information, Fig. S1b).

Finally, most *aub* mutant embryos fail to develop, although they are fertilized.^12,30^ To address whether the lack of Nos protein in *osk-OE; aub*^*−/−*^ embryos could result from their arrest of embryonic development, we quantified Nos protein levels in *osk-OE* unfertilized eggs that are activated by egg laying but do not develop. Nos levels were similar in *osk-OE* unfertilized eggs and embryos, demonstrating that the defect in Nos protein synthesis in *osk-OE; aub*^*−/−*^ embryos did not result from their lack of embryonic development (Supplementary information, Fig. S1c).

Together, these results show that piRNA-guided Aub binding to *nos* mRNA plays a direct role in translational activation in the presence of Osk.

### Ectopic expression of Osk leads to the formation of granules related to germ granules in the soma

**Fig. 2:**
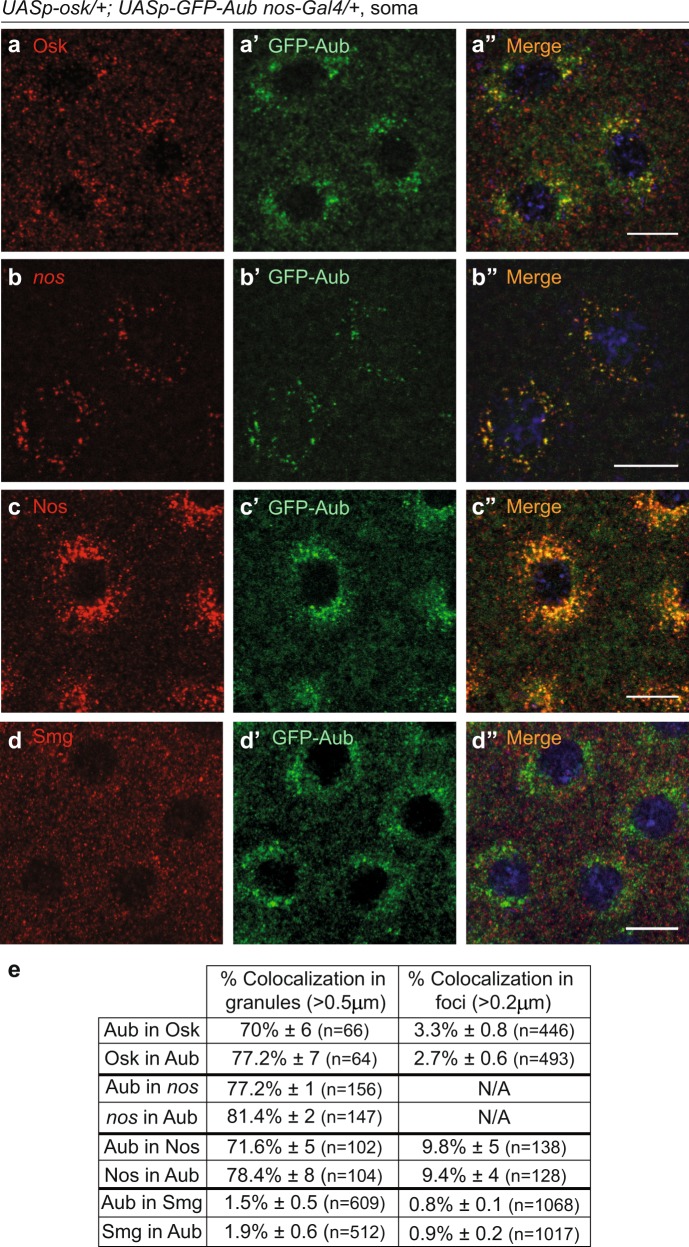
Ectopic expression of Osk nucleates RNA granules related to germ granules in the soma. **a**–**d”** Immunostaining of *UASp-osk/+; UASp-GFP-Aub nos-Gal4/+* embryos with anti-Osk (red) and anti-GFP (green) to visualize Aub (**a**–**a”**); anti-Nos (red) and anti-GFP (green) (**c**–**c”**); and anti-Smg (red) and anti-GFP (green) (**d**–**d”**); and smFISH of embryos with the same genotype revealing *nos* mRNA and GFP-Aub through GFP fluorescence (**b**–**b”**). DNA was visualized using DAPI. Scale bars, 10 μm. **e** Quantification of colocalization of immunostaining and smFISH shown in **a**–**d”**, using the Imaris software. Granules (> 0.5 μm) and foci (> 0.2 μm) were quantified around nuclei and in the cytoplasm between nuclei, respectively.

In the germ plasm, Osk leads to the assembly of germ granules that are large ribonucleoprotein particles containing mRNAs required for germ cell specification and development.^4,31^ In addition to Osk, Aub is a core component of germ granules.^32^ We asked whether Osk ectopic expression in the somatic part of the embryo could lead to the formation of RNA granules related to germ granules, containing Aub and *nos* mRNA. Immunostaining of *osk-OE* embryos also expressing GFP-Aub revealed that Osk was present in the bulk of the embryo where it accumulated in cytoplasmic foci that became larger around nuclei (Fig. 2a). GFP-Aub was also present in cytoplasmic foci in the bulk of *osk-OE* embryos and in larger foci around nuclei. Small foci of either Osk or GFP-Aub were dispersed in the cytoplasm and did not colocalize. However, Osk and GFP-Aub colocalized in larger foci that surrounded nuclei, indicating a different composition of these large foci (Fig. 2a–a”, e). smFISH of *nos* mRNA in embryos of the same genotype showed that *nos* mRNA accumulated in larger foci around nuclei where it colocalized with GFP-Aub (Fig. 2b–b”, e). Strikingly, Nos protein also accumulated around nuclei and partially colocalized with GFP-Aub in large foci, suggesting that *nos* mRNA translation occurred in the vicinity of these granules (Fig. 2c–c”, e). In contrast, in *osk-OE* embryos, Smg protein was present in foci that did not concentrate around nuclei and did not colocalize with large GFP-Aub foci (Fig. 2d–d”, e). This result was consistent with the reorganization of Smg into small foci in the germ plasm as compared to the somatic region in WT embryos, which suggested that Smg interaction with Osk did not take place within germ granules.^13^

We conclude that the presence of Osk in the somatic part of *osk-OE* embryos induces the formation of RNA granules that share functional similarities with germ granules, in which Aub and *nos* mRNA accumulate and at the proximity of which *nos* mRNA is translated.

### Aub interacts with translation initiation factors

**Fig. 3:**
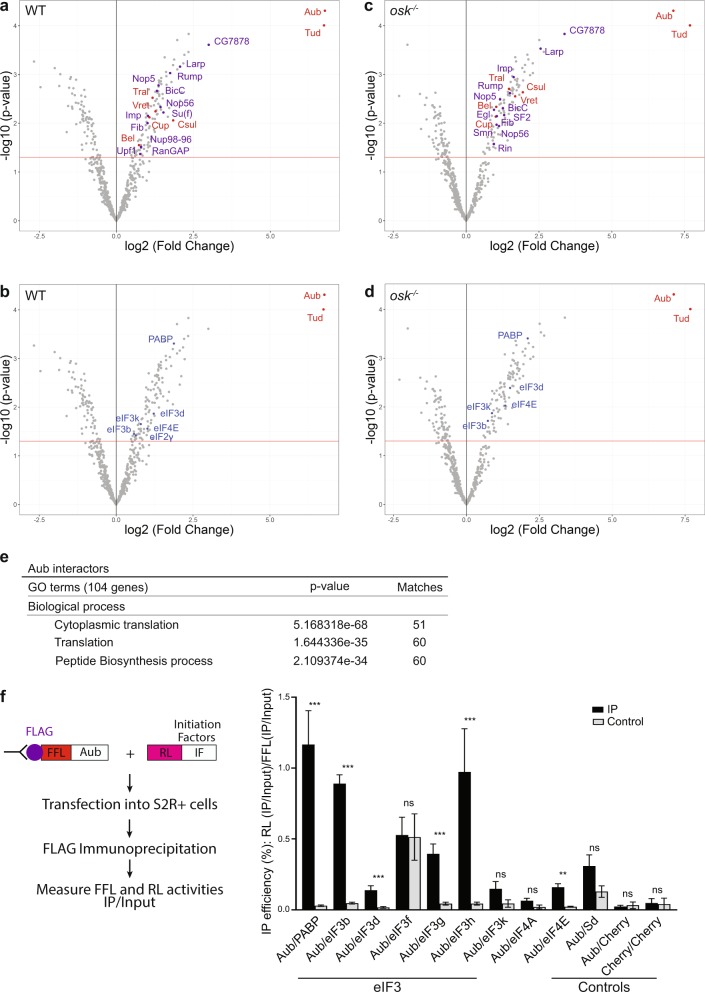
Identification of Aub-interacting partners. **a**–**d** Volcano plots showing the mass spectrometry analysis of GFP-Aub immunoprecipitation from 0–2 h embryos. Embryos expressing cytoplasmic GFP were used as control. *UASp-GFP-Aub nos-Gal4* embryos (**a**, **b**); *osk*^*54*^*; UASp-GFP-Aub/nos-Gal4* embryos (**c**, **d**). The analysis was based on four biological replicates. The red line indicates the significance threshold (*P* = 0.05). Known Aub interactors and RNA-binding proteins are indicated in red and purple, respectively (**a**, **c**); translation initiation factors are indicated in blue (**b**, **d**). **e** GO analysis of proteins identified as Aub interactors by mass spectrometry. **f** Validation of Aub interactors using the LUMIER assay. Left: schematic representation of the assay (FFL Firefly luciferase; RL Renilla luciferase). Right: graph plotting the IP efficiency of the indicated proteins. The values are IP efficiencies of the coprecipitation of the RL fusion proteins (IP/Input) normalized by the IP/Input values for FLAG-FFL-Aub. Error bars represent SD. Stars indicate values significantly greater than six times the mean value obtained in the control IPs without anti-FLAG antibody (Control). Scalloped (Sd) and Cherry proteins were used as negative controls. ***P < 0.001, **P < 0.01, ns not significant, using the *Z*-test.

To further decipher the function of Aub, we identified Aub interactors in embryos. GFP-Aub was immunoprecipitated from *UASp-GFP-Aub nos-Gal4* 0–2 h embryos and the coprecipitated proteins were analyzed using mass spectrometry. Embryos expressing GFP alone were used as negative controls (Supplementary information, Fig. S2a). 107 proteins were significantly enriched in GFP-Aub immunoprecipitation (IP) (*P* < 0.05) (Supplementary information, Table S1). Known Aub interactors were identified, including Tudor (Tud) that is restricted to the germ plasm and required for Aub accumulation in the germ plasm,^33,34^ three components of the *nos* translation repressor complex, Trailer hitch (Tral), Belle (Bel) and Cup,^35^ and Capsuleen/PRMT5 (Csul), the methyltransferase responsible for Aub arginine dimethylation^36^ (Fig. 3a). Several RNA-binding proteins were also found in GFP-Aub IP (Fig. 3a). Importantly, six translation initiation factors were identified as Aub interactors, among which are PABP, three subunits of eIF3 (eIF3d, eIF3k and eIF3b), and eIF4E, another component of *nos* translation repressor complex^35^ (Fig. 3b). In addition, 48 ribosomal proteins coprecipitated with Aub (Supplementary information, Fig. S2b). Gene Ontology (GO) term enrichment analysis using FlyMine (http://www.flymine.org) identified “Translation” as the most enriched term among Aub interactors (Fig. 3e). We also analyzed Aub interactors in *osk*^*54*^ mutant embryos that do not form germ plasm, with the aim of identifying specific Aub interactors in the germ plasm, which might be lost in *osk* mutant embryos. However, mass spectrometry of GFP-Aub IP from *osk*^*54*^ mutant embryos identified a very similar set of proteins to that identified in *osk*^*+*^ embryos (Fig. 3c, d; Supplementary information, Fig. S2c, Table S1). These data suggested that Osk might not affect Aub interaction with most of its protein interactors, but rather their activity. Indeed, PABP and eIF4E are found in the *nos* translational repressor complex, although they do not activate translation in this complex.^35^ eIF3 subunits were found to be in complex with Aub in the absence of Osk, suggesting that they might also be present in the *nos* repressed mRNP. The presence of Osk, by remodeling the mRNP, would allow to switch on their activity in translational activation.

We used quantitative luminescence-based coIP (LUMIER) assays to validate Aub interactions with translation initiation factors.^37^ Aub was fused to FLAG-tagged Firefly luciferase (FFL), whereas potential interactors were fused to Renilla luciferase (RL). Following transient expression in *Drosophila* S2R+ cells, Aub was immunoprecipitated with anti-FLAG antibodies, or without antibodies as negative control, and interactor coIP was quantified by recording Renilla and Firefly luciferase activities (Fig. 3f). PABP, four subunits of eIF3 (eIF3b, eIF3d, eIF3g and eIF3h) among six tested subunits, and eIF4E were found to significantly coprecipitate with Aub in these assays. Thus, although eIF3k that was identified as an Aub interactor by mass spectrometry could not be confirmed with the LUMIER assay, eIF3d and eIF3b interaction with Aub was confirmed, and two other eIF3 subunits, eIF3g and eIF3h were found to be in complex with Aub. Differences in the interaction between Aub and eIF3 individual subunits between embryos and S2R+ cells likely resulted from differences in these two experimental systems.

These results reveal that Aub physically interacts with the translation machinery and are consistent with a direct function of Aub in translation regulation.

### Aub interaction with PABP and eIF3d

**Fig. 4:**
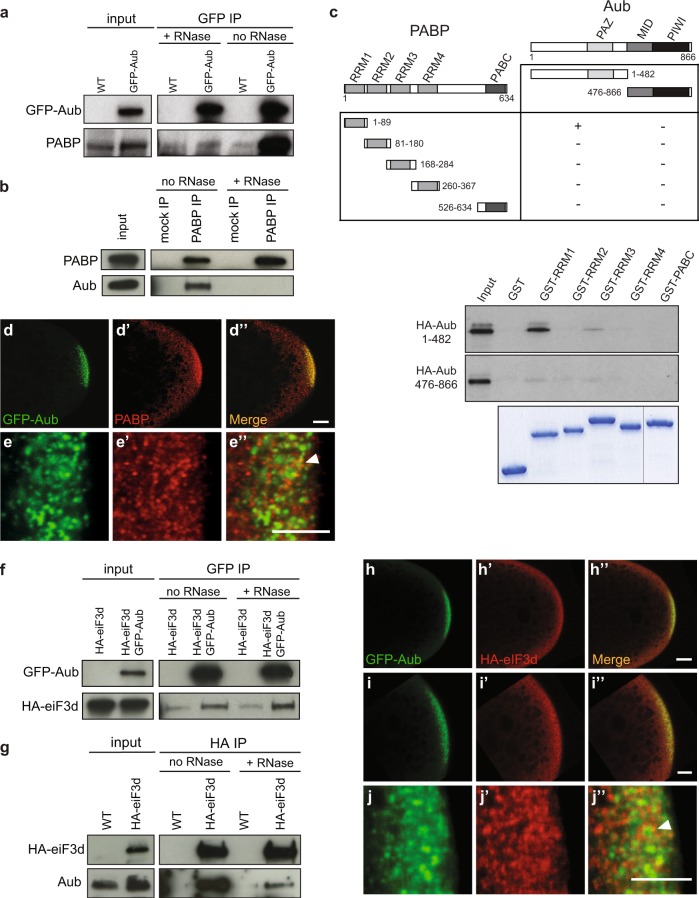
Aub physical interaction with PABP and eIF3d. **a**, **b** CoIP of PABP with GFP-Aub (**a**) and of Aub with PABP (**b**) in 0–2 h embryos. WT (mock IP) or *nos-Gal4 UASp-GFP-Aub* (GFP IP) embryo extracts were immunoprecipitated with anti-GFP, either in the absence or the presence of RNase A. Western blots were revealed with anti-GFP and anti-PABP (**a**). WT embryo extracts were immunoprecipitated with anti-PABP (PABP IP) or rabbit serum (mock IP), either in the absence or the presence of RNase A. Western blots were revealed with anti-PABP and anti-Aub (**b**). Inputs are extracts before IP in **a** and **b**. **c** GST pull-down assays between GST-PABP and HA-Aub. Constructs and interactions are shown in the table. HA-tagged Aub fragments were revealed using western blot with anti-HA. Inputs correspond to 1/10 of in vitro synthesized HA-Aub fragments before pull-down. GST alone was used as negative control. GST and GST-recombinant proteins used in each pull-down are shown in the bottom gel. **d**–**e”** Immunostaining of *UASp-GFP-Aub nos-Gal4* embryos with anti-GFP (green) to visualize Aub and anti-PABP (red). Posterior of embryos are shown. Higher magnification showing the distribution of Aub-containing germ granules and PABP foci (**e**–**e”**). Colocalization and overlap between Aub and PABP staining are quantified in Supplementary information, Fig. S3a. The white arrowhead shows PABP foci surrounding a germ granule. Scale bars, 20 μm in **d** and 5 μm in **e**. **f**, **g** CoIP of HA-eIF3d with GFP-Aub (**f**) and of Aub with HA-eIF3d (**g**) in 0–2 h embryos. *UASp-HA-eIF3d/+; nos-Gal4/+* (mock IP) or *UASp-HA-eIF3d/+; UASp-GFP-Aub nos-Gal4/+* (GFP IP) embryo extracts were immunoprecipitated with anti-GFP, either in the absence or the presence of RNase A. Western blots were revealed with anti-GFP and anti-HA (**f**). WT (mock IP) or *UASp-HA-eIF3d/+; nos-Gal4/+* (HA IP) embryo extracts were immunoprecipitated with anti-HA, either in the absence or the presence of RNase A. Western blots were revealed with anti-HA and anti-Aub (**g**). Inputs are extracts before IP in **f** and **g**. **h**–**j”** Immunostaining of *UASp-HA-eIF3d/+; UASp-GFP-Aub nos-Gal4/+* embryos with anti-GFP (green) to visualize Aub and anti-HA (red) to visualize eIF3d. Posterior of embryos are shown. Higher magnification showing the slight accumulation of HA-eIF3d at the posterior pole (**i**–**i”**), and the distribution of Aub-containing germ granules and eIF3d foci (**j**–**j”**). Colocalization and overlap between Aub and eIF3d staining are quantified in Supplementary information, Fig. S3d. The white arrowhead shows eIF3d foci surrounding a germ granule. Scale bars, 20 μm in **h**, 10 μm in **i** and 5 μm in **j**.

Because PABP and eIF3d showed the strongest association with Aub in the mass spectrometry analysis, and have key roles in translation initiation, we further investigated their interaction with Aub. We used coIP to address Aub physical interaction with PABP in embryos. PABP coprecipitated with GFP-Aub in 0–2 h embryos; however, this coprecipitation was strongly reduced in the presence of RNase (Fig. 4a). In the reverse experiment, PABP was also able to coprecipitate Aub, but this coprecipitation was abolished in the presence of RNase (Fig. 4b). These results could indicate either that Aub and PABP did not interact directly and coprecipitated through their binding to the same mRNAs, or that Aub direct interaction with PABP was stabilized by mRNA in a tripartite association. To address this question, we analyzed direct interaction between Aub and PABP using GST pull-down assays. Aub has three domains characteristic of Argonaute proteins (PAZ, MID and PIWI) and was separated into two parts, Aub (1–482) that contains the N-terminal and PAZ domains, and Aub (476–866) that contains the MID and PIWI domains (Fig. 4c). PABP is composed of four RNA recognition motifs (RRM1-4), a proline-rich linker region and a PABP C-terminal (PABC) domain.^38^ Each RRM and the PABC domain were fused separately to GST. In vitro-translated HA-tagged Aub(1–482) bound to recombinant GST-RRM1, but not to the other PABP domains fused to GST or GST alone. In addition, HA-Aub (476–866) did not bind to any PABP domain (Fig. 4c). These data revealed direct interaction between RRM1 of PABP and the N-terminal half of Aub. They were consistent with the model in which Aub directly interacted with PABP and mRNA stabilized this interaction in embryos.

We then analyzed potential colocalization of Aub and PABP. Co-immunostaining of GFP-Aub-expressing embryos with anti-PABP and anti-GFP antibodies showed that PABP was distributed in the whole embryo and specifically accumulated in the germ plasm (Fig. 4d–d”). Strikingly, PABP was present in foci, and in the germ plasm a large proportion of Aub-containing germ granules (79.4%, Supplementary information, Fig. S3a) either colocalized with, or were in close proximity to and surrounded by PABP foci, with a partial overlap of both proteins (Fig. 4e–e”; Supplementary information, Fig. S3a).

eIF3 is composed of twelve subunits and one associated factor, and coordinates several steps of translation initiation.^39^ Interestingly, in addition to this role in basal translation, eIF3 plays regulatory roles in the translation of specific mRNAs. eIF3d appears to be a major actor in eIF3 regulatory functions, either through its binding to 5′UTR of specific mRNAs, leading to cap-independent translation, or directly through its interaction with the cap structure.^40,41^ Aub interaction with eIF3d was analyzed in embryos using coIP. GFP-Aub was able to coprecipitate HA-eIF3d in 0–2 h embryos, and this coprecipitation was maintained in the presence of RNase (Fig. 4f). Conversely, HA-eIF3d was able to coprecipitate Aub in 0–2 h embryos, and although less efficient, this coprecipitation remained in the presence of RNase (Fig. 4g). Colocalization of Aub and eIF3d was analyzed in embryos expressing both GFP-Aub and HA-eIF3d. eIF3d was present in the whole embryo with a slight accumulation in the germ plasm (Fig. 4h–i”; Supplementary information, Fig. S3b, c). Similarly to PABP, eIF3d formed foci, and in the germ plasm most Aub-containing germ granules (83.2%, Supplementary information, Fig. S3d) colocalized with or were surrounded by eIF3d foci, with a partial colocalization of both proteins at the edge of the granules (Fig. 4j–j”; Supplementary information, Fig. S3d).

Taken together, these results show that Aub is in complex with the translation initiation factors PABP and eIF3d. In the germ plasm, PABP and eIF3d have a specific organization around germ granules and colocalize with Aub at the periphery of the granules, suggesting that translation might take place at the edge of germ granules.

### Mechanism of Aub-dependent translational activation

**Fig. 5:**
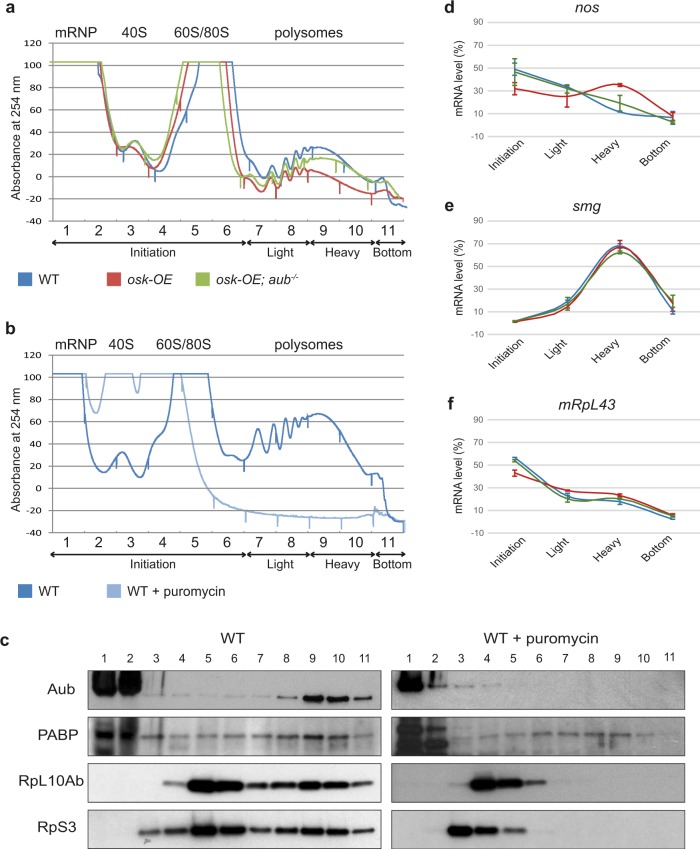
Aub acts at the level of translation initiation. **a** Profile of absorbance at 254 nm for 0–2 h WT (blue), *osk-OE* (red) and *osk-OE; aub*^*–/–*^ (green) embryo extracts fractionated into 10%–50% sucrose gradients. Complete genotypes are as in Fig. 1. Fractions were pooled as indicated on the graph into: initiation (fractions 1–6), light polysomes (fractions 7 and 8), heavy polysomes (fractions 9 and 10) and bottom (fraction 11). **b** Profile of absorbance at 254 nm for 0–2 h WT embryos treated (light blue), or not (dark blue) with puromycin, fractionated into 10%–50% sucrose gradients. **c** Western blot showing the distribution of Aub and PABP through the gradient from WT embryos treated or not with puromycin. Two ribosomal proteins, RpL10Ab (60S ribosome subunit) and RpS3 (40S ribosome subunit) were used to record puromycin treatment efficacy. **d**–**f** Quantification of *nos* (**d**), *smg* (**e**) and *mRpL43* (**f**) mRNAs using RT-qPCR in the different fractions of the gradients for WT (blue), *osk-OE* (red) and *osk-OE; aub*^*–/–*^ (green) embryos. mRNA levels are indicated in percentage of total mRNA in all the fractions. Mean of two biological replicates, quantified in triplicate. Error bars represent SEM. *smg* and *mRpL43* were used as control mRNAs.

Aub association with translation initiation factors suggested that Aub might activate *nos* mRNA translation at the level of initiation. We directly addressed this question using polysome profiling in which mRNA-protein complexes are separated by fractionation through linear sucrose gradients.^42^ mRNA localization within the sucrose gradient reflects its translation status: migration in the light RNP or monosomal fractions of the gradient indicates a lack of translation, whereas migration in the heavy polysomal fractions indicates active translation. Polysome profiling was performed with 0–2 h WT, *osk-OE* and *osk-OE; aub*^*−/−*^ embryos. The abundance of polysomes was reduced in *osk-OE* embryos compared to WT, indicating that ectopic expression of Osk in the whole embryo affected basal translation (Fig. 5a). In contrast, polysome abundance was partially restored in *osk-OE; aub*^*−/−*^ embryos, revealing that translation was active in these embryos (Fig. 5a). Thus, the level of basal translation was affected oppositely to the level of Nos protein. This is consistent with Aub being involved in a regulatory mode of translation occurring on specific mRNAs. To confirm this point, we used *smg* mRNA as a control, since it is highly translated in the whole embryo upon egg activation.^43,44^ Smg protein levels were not decreased in *osk-OE; aub*^*−/−*^ embryos compared to *osk-OE* embryos, confirming the specificity of Aub-dependent translational activation (Supplementary information, Fig. S4a). Western blot analysis of the gradient fractions revealed co-sedimentation of Aub with actively translating mRNAs in the heavy polysomal fractions, and the presence of PABP in these fractions (Fig. 5b, c). To confirm Aub association with actively translating mRNAs, we treated embryo lysates with puromycin that causes premature termination of elongating ribosomes. Puromycin treatment efficiency was validated by the complete disassembly of polysomes visualized by absorbance measurement of OD at 254 nm, and the shift of ribosomal proteins to monosomal and lighter fractions containing 60S and 40S ribosomal subunits (Fig. 5b, c). Aub shifted to the light mRNP fractions in the presence of puromycin, indicating its *bona fide* association with translating mRNAs. In contrast, although PABP was shifted towards lighter fractions of the gradients in the presence of puromycin, a certain amount remained present in most fractions, suggesting the presence of heavy RNA complexes containing PABP in *Drosophila* embryos (Fig. 5c). This is consistent with the presence of mRNAs in heavy fractions of sucrose gradients independently of translation, in polysome gradients from early embryos.^45^ We then quantified mRNA through polysome gradients using RT-qPCR. *nos* mRNA was mostly present in initiation and light polysomal fractions in WT embryos, in agreement with a low amount of *nos* mRNA being actively translated (Fig. 5d). In *osk-OE* embryos, the level of *nos* mRNA decreased in the initiation fractions whereas it increased in the heavy polysomal fractions, consistent with the 2-fold increase of Nos protein levels in these embryos (Figs. 1a, b, 5d). Quantification of *nos* mRNA through the gradient in the presence of puromycin confirmed that the pool of *nos* present in the heavy fractions was indeed associated with actively translating polysomes (Supplementary information, Fig. S4b–d). Interestingly, in *osk-OE; aub*^*−/−*^ embryos, the distribution of *nos* mRNA was similar to that in WT embryos, with higher amounts of mRNA in initiation fractions and lower amounts in heavy polysomal fractions (Fig. 5d). These results suggested the role of Aub at the initiation step of translation. To further confirm the role of Aub in translational activation of specific mRNAs, we quantified *smg* and *mRpL43* mRNAs through the polysome gradients. Consistent with *smg* active translation in early embryos, most *smg* mRNA was present in heavy polysomal fractions, and this profile was not affected in *osk-OE* and *osk-OE; aub*^*−/−*^ embryos, indicating that *smg* translation was independent of both Osk and Aub (Fig. 5e). *mRpL43* was used as a control mRNA that is not bound by Aub^12^ and similarly, its distribution through the gradient was not strongly affected in *osk-OE* and *osk-OE; aub*^*−/−*^ embryos (Fig. 5f).

These results show that Aub plays a role in the translation of specific mRNAs and are consistent with Aub acting at the level of translation initiation.

### eIF3d plays a role in Aub-dependent translational activation

**Fig. 6:**
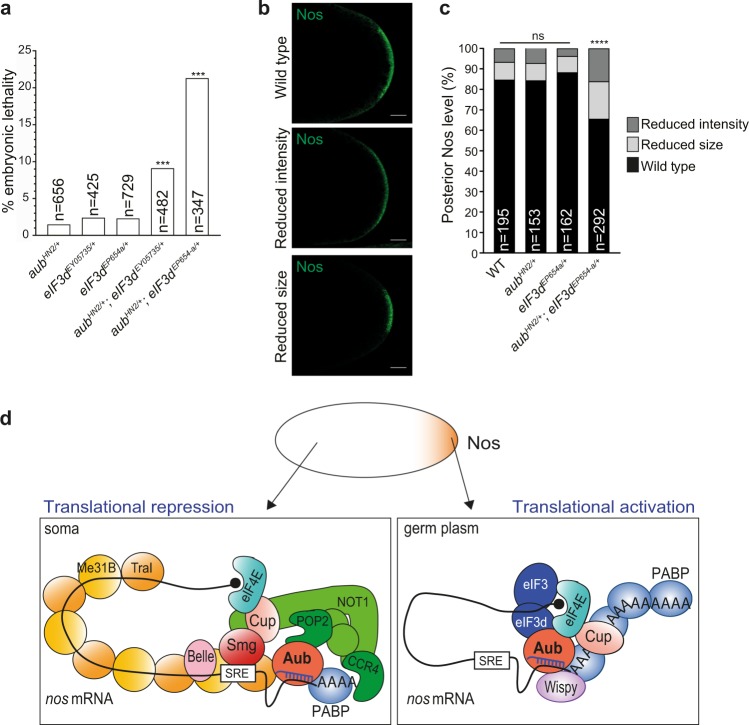
Aub and eIF3d functionally interact for *nos* mRNA translation. **a** Percentage of embryonic lethality of single or double *aub* and *eIF3d* heterozygous mutants. The genotypes are indicated. ***P < 0.001, using the χ^2^ test. **b** Immunostaining of single and double *aub* and *eIF3d* heterozygous mutant embryos with anti-Nos antibody. Posterior of embryos with the three types of staining: wild type, reduced size or reduced intensity, are shown. Scale bars, 20 μm. **c** Quantification of posterior staining shown in **b** using the ImageJ software. For each genotype, the percentage of embryos with each staining category was recorded. ****P < 0.0001, ns not significant, using the χ^2^ test. **d** Model of Aub-dependent translational activation. In the somatic part of the embryo, *nos* mRNA translation is repressed by two mechanisms: a cap-dependent mechanism that involves Cup binding to eIF4E, and a cap-independent mechanism that involves the coating of the mRNA by Me31B and Tral. Both mechanisms might depend on the CCR4-NOT complex recruited by Smg and Aub. In the germ plasm, Smg binding to Osk precludes its interaction with *nos* mRNA, leading to depletion of CCR4-NOT and remodeling of the mRNP. This would lead to the dissociation of Me31B/Tral from the mRNA. Aub interaction with PABP and eIF3 subunits would allow unconventional translation, bypassing eIF4E requirement. The recruitment of Wispy poly(A) polymerase by Aub leading to polyadenylation is likely to also contribute to translation activation. Note that eIF3 might be present in the repressor complex in the soma, since eIF3 was found as Aub interactor in *osk* mutant embryos; however, its activity in translation activation would be repressed.

To address the biological relevance of Aub/eIF3d physical interaction, we analyzed the effect of the concomitant reduction of *aub* and *eIF3d* gene dosage by half. Although single *aub* or *eiF3d* heterozygous mutant embryos showed a low level of lethality (2%–3%), embryonic lethality significantly increased up to 21% in double heterozygous mutants, suggesting that Aub and eIF3d act together in embryonic development (Fig. 6a). *nos* mRNA translation was then recorded in these embryos using immunostaining. The Nos protein level visualized by immunofluorescence at the posterior cortex was quantified. In WT, 85% of embryos showed a full accumulation of Nos protein at the posterior pole, whereas 15% had a reduced accumulation (Fig. 6b, c). Nos accumulation in heterozygous *aub* or *eIF3d* mutant embryos was similar to that in WT. In contrast, in *aub*^*–/+*^*; eIF3d*^*–/+*^ double heterozygous mutants, the percentage of embryos with reduced Nos accumulation significantly increased to 34% (Fig. 6b, c; Supplementary information, Fig. S5a). This reduction of Nos accumulation in double heterozygous mutants did not correlate with reduced Osk accumulation or reduced *nos* mRNA localization at the posterior pole (Supplementary information, Fig. S5b–e), indicating a direct defect in *nos* mRNA translation. We conclude that Aub/eIF3d physical interaction is required for *nos* mRNA translational activation.

## Discussion

Several studies have reported the role of PIWI proteins in cellular mRNA regulation at the level of stability. piRNA-dependent binding of mRNAs by PIWI proteins leads to their decay in different biological systems.^16^ In addition, in *Drosophila* embryos, mRNA binding by the PIWI protein Aub also leads to their stabilization in a spatially regulated manner.^13^ Here, we report a novel function of Aub in direct translational control of mRNAs. Using *nos* mRNA as a paradigm, we show that Aub is required for *nos* mRNA translation. Nos protein levels are also strongly reduced in *armi* mutant, in which piRNA biogenesis is massively affected,^27^ suggesting that Aub loading with piRNAs is necessary for its function in translational activation. Consistent with this, we find that deletion of two piRNA target sites in *nos* mRNA decreases its translation. Importantly, Nos levels are not affected in a *panx* mutant background. Panx is a piRNA factor required for transcriptional repression of transposable elements, but has no function in piRNA biogenesis.^28,29^ In addition, as is the case for *aub* and *armi* mutants, *panx* mutant embryos do not develop.^28,29^ Finally, Nos levels are similar in unfertilized eggs and embryos overexpressing Osk, demonstrating that Nos protein synthesis is independent of embryonic development. Together, these results strongly argue for a direct role of Aub and piRNAs in *nos* mRNA translational control, independently of their role in transposable element regulation or developmental defects in piRNA pathway mutants.

Mass spectrometry analysis of Aub interactors points to a strong link with the translation machinery. In addition, polysome gradient analyses reveal Aub association with actively translated mRNAs in polysomal fractions. A link has been reported previously between the PIWI proteins Miwi and Mili and the translation machinery in mouse testes, where Miwi and Mili were found to associate with the cap-binding complex.^46,47^ However, the role of Miwi and Mili in translational control has not been characterized. We now decipher the molecular mechanisms of Aub function in translational activation of germ cell mRNAs in the *Drosophila* embryo. We demonstrate a physical interaction between Aub and the translation initiation factors PABP, eIF4E and subunits of the eIF3 complex. These interactions are in agreement with polysome gradient analyses in WT and *aub* mutant backgrounds that indicate a role of Aub in translation initiation.

Recent data have identified specific roles of eIF3 in the regulation of translation. eIF3 is the most elaborate of translation initiation factors containing twelve subunits and an associated factor, eIF3j. This complex promotes all steps of translational initiation and does so in part through direct association with other translation initiation factors, contributing to their functional conformations on the small ribosomal subunit surface.^39^ In addition to this role in basal translation, the eIF3a, b, d and g subunits were shown to directly bind 5′UTR of specific mRNAs, leading to cap-dependent translation activation or repression.^40^ The eIF3d subunit that attaches to the edge of the complex appears to play an especially important role in various modes of eIF3-dependent translational control: (1) eIF3d is involved in the translational repression of *Drosophila sex-lethal* mRNA through binding to its 5′UTR.^48^ (2) eIF3d was reported to directly bind the cap structure of specific mRNAs in mammalian cells, thus bypassing the requirement of eIF4E binding to the cap for translation initiation.^41^ (3) In the same line, eIF3d was involved in cap-dependent translational activation of specific mRNAs for neuronal remodeling in *Drosophila* larvae, in a context where eIF4E is blocked by 4E-binding protein (4E-BP).^49^ Other studies have reported the role of eIF3 in promoting cap-independent translation, thus highlighting eIF3 functional versatility in the control of translation. eIF3 was shown to directly bind methylated adenosine m^6^A, in mRNA 5′UTRs to induce cap-independent translation under stress conditions.^50^ Furthermore, PABP bound to the poly(A) tail was also shown to cooperate with eIF3 for its binding to mRNA 5′UTR triggering cap-independent translation.^51^

Here, we described a new mode of eIF3-dependent translational activation through its recruitment by the PIWI protein Aub. Based on previous information on the *nos* translation repressor complex and data presented here on translational activation, we propose the following model (Fig. 6d). *nos* mRNA translation is repressed in the somatic part of the embryo by two mechanisms.^11,35^ First, the 4E-BP protein Cup in complex with Smg binds to eIF4E and prevents eIF4G recruitment and cap-dependent translation.^11,52^ The detailed mechanism of Cup recruitment to the repressor complex has not been clarified, but Cup was shown to directly associate with the Not1 subunit of the CCR4-NOT complex and this interaction might stabilize Cup association with eIF4E.^53^ CCR4-NOT itself is recruited to *nos* mRNA by Smg and Aub.^9,18^ Second, two translational repressors, the RNA helicase Me31B (*Drosophila* DDX6) and its partner Tral coat the length of *nos* mRNA and prevent translation through a cap-independent mechanism.^35^ Again the mode of Me31B/Tral specific recruitment to *nos* mRNA has not been determined, but the CCR4-NOT complex might also be involved since DDX6 directly binds the Not1 subunit of CCR4-NOT.^54,55^ Aub coprecipitation with components of the *nos* translational repressor complex is consistent with its association with the CCR4-NOT complex in the soma^18^ and suggests that Aub might be involved in translational repression, in addition to mRNA decay. In the germ plasm, Osk interaction with Smg prevents Smg binding to *nos* mRNA^9^ and this contributes to CCR4-NOT displacement from the mRNP complex. Consistent with this, CCR4 is depleted in the germ plasm.^13^ The lack of CCR4-NOT on *nos* mRNA might preclude the recruitment of Me31B/Tral and relieve the cap-independent mechanism of translational repression (Fig. 6d). We find that Aub physically interacts with PABP and several subunits of eIF3. We propose that these associations would lead to translational activation independently of eIF4E through binding of eIF3 to *nos* 5′UTR, followed by direct recruitment of the 40 S ribosome by eIF3 and PABP, as previously reported for translation of *XIAP* mRNA.^51^ Alternatively, eIF3 might act through direct binding of eIF3d to the cap structure; however, we do not favor this hypothesis. Indeed, if eIF3d interaction with the cap was involved, overexpression of the point mutant eIF3d^helix11^ that is unable to bind the cap,^41^ would be expected to induce negative dominant defects, due to the lack of translation mediated by this interaction.^49^ However, overexpression of eIF3d^helix11^ with the *nos-Gal4* driver did not induce any defects in embryonic development or Nos protein synthesis (Supplementary information, Fig. S6).

Germ granules coordinate germ cell mRNA regulation with piRNA inheritance through the role of PIWI proteins in both processes. Recent studies in *C. elegans* have shown that piRNA/PRG1-dependent mRNA accumulation in germ granules prevent their silencing, strengthening the function of piRNAs in germ granules for mRNA storage and surveillance.^56,57^ In *Drosophila*, Aub mediates the link between piRNAs and mRNA regulation in germ granules since Aub localization to germ granules depends on its loading with piRNAs^12^ and Aub/piRNAs play a general role in the localization and stabilization of germ cell mRNAs in germ granules.^13,19^ How do germ granules accommodate translational control has remained more elusive. In *Drosophila* embryos, germ granules contain mRNAs that are translated sequentially.^58^ We demonstrate a direct role of Aub in translational activation. Strikingly, PABP and eIF3d tend to colocalize with Aub at the periphery of germ granules. This is reminiscent of a study analyzing translational control in relation to RNA granules in *Drosophila* oocytes, in which translational repressors such as Me31B were found to concentrate in the granule core with repressed mRNAs, whereas the translational activator Orb was localized at the edge of the granules where mRNAs docked for translation.^59^ Similarly, germ granules in embryos might be partitioned into functional subdomains involved in various steps of mRNA regulation, including storage (in an internal region of granules) and translational activation (at the granule periphery). Our work reveals the central role of Aub in activation of translation. Future studies will undoubtedly address the complexity of mRNA regulation by PIWI proteins in relation with germ granules.

While this manuscript was under review, a role of Miwi and piRNAs in translational activation during mouse spermiogenesis has been demonstrated.^60^ Miwi was shown to be in complex with PABP and several subunits of eIF3 for its function in translational activation, which is required for spermatid development. This reveals a striking evolutionary conservation of PIWI protein function in translational control for key developmental processes.

## Materials and methods

### *Drosophila* lines

*w*^*1118*^ was used as a control. Mutant alleles and transgenic lines were *aub*^*QC42*^ *cn*^*1*^*bw*^*1*^*/CyO* and *aub*^*HN2*^*cn*^*1*^*bw*^*1*^*/CyO*,^61^ *nos-Gal4-VP16*,^62^ *UASp-osk-K10*,^25^ *panx*^*M1*^ and *panx*^*M4*^,^29^ *armi*^*1*^,^63^ *armi*^*72.1*^,^64^ *nos*^*BNx*^,^65^ *nos(ΔpirooΔpi412); nos*^*BN*^*/TM3 Sb*,^13^ *w; osk*^*54*^ *nos-Gal4-VP16/TM3 Sb* and *yw; osk*^*54*^ *e UASp-GFP-Aub/TM3 Sb*,^12^ *UASp-GFP-Aub*,^66^ *UAS-GFP cytoplasmic* (gift from J.M. Dura), *eIF3d*^*EY05735*^ (#20072) and *eIF3d*^*EP-654a*^ (#43437) (Bloomington *Drosophila* Stock Center). The *UASp-HA-eIF3d* and *UASp-HA-eIF3d*^helix11^ lines were generated in this study by insertion of PhiC31 recombination into attP40 site (BestGene). The genotypes of embryos (aged 0–2 h) indicated throughout were the genotypes of mothers. Females of the indicated genotypes were crossed with WT males.

### S2R+ cells

S2R+ cells (Gift from G. Cavalli) were cultivated at 25 °C in Schneider medium complemented with 10% fetal bovine serum (Gibco) and 1% penicillin-streptomycin (Gibco).

### Immunostaining and image analysis

0–2 h embryos were collected in a basket from plates, washed in tap water and dechorionated using commercial bleach for 2 min, rinsed and dried. Embryos were then fixed at the interface of a 1:1 solution of 36% formaldehyde:100% heptane for 5 min, followed by 100% methanol devitellinization. Embryos were re-hydrated, blocked in 1% BSA for 1 h and incubated overnight with primary antibodies. Secondary antibody incubation, after washes in PBS, 0.1% Tween, was performed for 1 h at room temperature. Embryos were mounted in Vectashield (Vector Laboratories) for imaging. Antibodies used: rabbit anti-Osk (1:1000, gift from P. Lasko), rabbit anti-Nos (1:1000, gift from A. Nakamura), rabbit anti-PABP (1:500, gift from A. Vincent), rabbit anti-Smg (1:2000),^67^ mouse anti-HA (1:2000, ascites produced from clone 12CA5), rabbit anti-GFP (1:1000, Invitrogen), mouse anti-GFP (1:1000, Roche), goat anti-mouse IgG Cy3 (1:1000, Jackson ImmunoResearch), goat anti-rabbit IgG Alexa-488 (1:800, Invitrogen) and donkey anti-rabbit Cy3 (1:1000, Jackson ImmunoResearch). Microscopy was performed using a Leica SP8 confocal scanning microscope. Data were processed and analyzed using the ImageJ software.

### smFISH

Dechorionated embryos were fixed at the interface of a 1:1 solution of 10% formaldehyde:100% heptane for 20 min, followed by 100% methanol devitellinization. After permeabilization in ethanol, embryos were washed 4 times for 15 min in PBT and then once for 20 min in Wash Buffer (10% 20× SCC, 10% formamide). They were then incubated overnight at 37 °C in Hybridization Buffer (10% formamide, 10% 20× SSC, 400 µg/mL tRNA, 5% Dextran sulfate, 1% VRC (Vanadyl Ribonucleoside Complexes, Sigma)) with anti-*nos* probes (Supplementary information, Table S2) coupled to CAL Fluor Red 590 (Stellaris). Embryos were washed in Wash Buffer at 37 °C and then in 2× SCC, 0.1% Tween at room temperature before mounting (Pro-Long Gold antifade reagent, Invitrogen). Microscopy was performed using a Leica SP8 confocal scanning microscope. Data were processed and analyzed using the ImageJ software.

### RNA extraction and RT-qPCR

Total RNA was prepared from 30 embryos using Trizol (Invitrogen) following recommendations from the manufacturer. For RT-qPCR, 1 μg of total RNA was reverse transcribed using Superscript III (Invitrogen) and random hexamers. Quantitative PCR (qPCR) was performed on a LightCycler LC480 (Roche) with Lightcycler 480 SYBR green master (Roche) and primers are listed in Supplementary information, Table S3. Quantifications were performed in triplicate.

### Coimmunoprecipitation and western blot

GFP immunoprecipitations for mass spectrometry were performed as follows: 0.5 g of 0–2 h-dechorionated embryos were crushed in DXB buffer (25 mM HEPES, 250 mM sucrose, 1 mM MgCl_2_, 1 mM DTT, 150 mM NaCl, protease inhibitor) with 0.1% Triton X-100 and RNasin and incubated on ice for 30 min. Lysates were centrifuged for 10 min and the supernatant was transferred to a new tube. Lysates were incubated on equilibrated GFP-trap beads (Chromotek) overnight at 4 °C on a wheel. Beads were washed seven times in DXB buffer complemented with 1% Triton X-100 and RNasin. Beads were suspended in 2× NuPAGE Blue supplemented with 50 mM DTT and incubated for 10 min at 95 °C. The quality of the samples was assessed by silver staining (SilverQuest, Invitrogen). For coIP experiments, 0.15–0.18 mg of 0–2 h embryos were crushed in IP buffer (20 mM Tris, pH 7.5, 150 mM NaCl, 0.2% NP-40, 1.5 mM DTT, 10 mM EGTA, protease inhibitor) with either 40 U/µL RNase A or 100 U/µL RNase inhibitor. Extracts were centrifuged at 10,000× *g* for 10 min at 4 °C and incubated on pre-equilibrated magnetic beads with anti-GFP (Chromotek) or anti-HA (Pierce) antibody for 2.5 h at 4 °C. After incubation, the beads were washed five times with IP buffer and immunoprecipitated proteins were eluted from beads by incubation with 2× Laemmli buffer supplemented with 10% β-mercaptoethanol for 5 min at 95 °C. Samples were then analyzed by western blot. For western blot analysis, protein extracts obtained from 30 embryos crushed in 30 µL of 2× Laemmli buffer supplemented with 10% β-mercaptoethanol were boiled for 5 min at 95 °C. Samples were then loaded onto 10% SDS-PAGE gels before transfer to a nitrocellulose membrane. The membrane was blocked for 1 h in 5% milk diluted in 1× PBS, 0.1% Tween 20 before proceeding to primary antibody incubation (overnight, 4 °C on a rotating plate). Antibodies and dilutions for western blot were: rabbit anti-Nos (1:1000, gift from A. Nakamura), rabbit anti-Osk (1:2000, gift from P. Lasko), mouse anti-Aub (4D10, 1:5000),^68^ guinea pig anti-Smg (1:2000, gift from C. Smibert), rabbit anti-PABP (1:500, gift from A. Vincent), rabbit anti-GFP (1:1000, Invitrogen), anti-HA (1:1000, Covance) and mouse anti-α-Tubulin (1:5000, Sigma). After washes in 1× PBS, 0.1% Tween 20, the membrane was incubated for 1 h at room temperature with secondary antibody coupled with HRP (Jackson ImmunoResearch). After washes, HRP-conjugated secondary antibodies were revealed by chemiluminescent detection (Pierce). Quantifications were performed with the ImageJ software using the Gels tool.

### Mass spectrometry

Total protein elute was loaded on 10% SDS-PAGE gels (Mini-Protean TGX Precast gels, Bio-Rad). For each sample, one band was cut after stacking migration. Gel pieces were destained with three washes in 50% acetonitrile and 50 mM TEABC (trimethy ammonium bicarbonate buffer). After protein reduction (10 mM DTT in 50 mM TEABC at 60 °C for 30 min) and alkylation (55 mM iodoacetamide in TEABC at room temperature in the dark for 30 min), proteins were in-gel digested using 1 µg Trypsin (Trypsin Gold, Promega). Digested products were dehydrated in a vacuum centrifuge. Obtained peptides were analyzed online using Q-Exactive Plus mass spectrometer (Thermo Fisher Scientific) interfaced with a nano-flow HPLC (RSLC U3000, Thermo Fisher Scientific). Samples were loaded onto a 15 cm reverse phase column (Acclaim Pepmap 100, NanoViper, Thermo Fisher Scientific) and separated using a 103-min gradient of 2%–40% of buffer B (80% acetonitrile, 0.1% formic acid) at a flow rate of 300 nL/min. MS/MS analyses were performed in a data-dependant mode (Xcalibur software 4.1, Thermo Fisher Scientific). Full scans (375–1500 m/z) were acquired in the Orbitrap mass analyzer with a 70,000 resolution at 200 m/z. The twelve most intense ions (charge states ≥ 2) were sequentially isolated and fragmented by HCD (high-energy collisional dissociation) in the collision cell and detected at 17,500 resolution. The spectral data were analyzed using the Maxquant software (v1.5.5.1) with default settings.^69^ All MS/MS spectra were searched by the Andromeda search engine against a decoy database consisting of a combination of *Drosophila melanogaster* entries from Reference Proteome (UP000000803, release 2018_02, https://www.uniprot.org/), isoform C sequence of Aub protein and classical contaminants, containing forward and reverse entries. Default search parameters were used; Oxidation (Met) and Acetylation (N-term) as variable modifications and Carbamidomethyl (Cys) as fixed modification were applied. FDR was set to 1% for peptides and proteins. A representative protein ID in each protein group was automatically selected using in-house bioinformatics tool (Leading_v3.2). First, proteins with the most numerous identified peptides are isolated in a “match group” (proteins from the “Protein IDs” column with the maximum number of “peptide counts”). For the match groups where more than one protein ID are present after filtering, the best annotated protein in UniProtKB, release 2019_01 (reviewed entries rather than automatic ones, highest evidence for protein existence) is defined as the “leading” protein. Label free quantification (MaxQuant LFQ) was used to identify differential proteins between samples.

### LUMIER assays

S2R+ cells (250,000) were transfected using Effectene transfection reagent (Qiagen) and incubated for 48 h at 25 °C. Cells were lysed in HNTG buffer (20 mM HEPES, 150 mM NaCl, 1 mM MgCl_2_, 1 mM EGTA, 1% Triton, 10% glycerol) complemented with protease inhibitor (cOmplete^TM^ EDTA-free Protease Inhibitor Cocktail, Roche). On a plate (LUMITRAC 600 96 W Microplate High Binding, Greiner) pre-coated with anti-FLAG antibody (Sigma, F1804) and blocked for 1 h with a blocking solution (3% BSA, 5% sucrose and 0.5% Tween 20), lysates were incubated for 3 h on ice. After washes with HNTG, luminescence was revealed using Dual-Luciferase Reporter Assay System (Promega, E1910) and read on a luminometer Tristar LB941. Transfections were repeated 8–32 times.

### Polysome profiling

For lysis of embryos, we used either of two methods that produce similar results. Either 0.2 g of fresh 0–2 h embryos were homogenized in lysis buffer composed of 20 mM Tris-HCl, 140 mM KCl, 5 mM MgCl_2_, 0.5 mM DTT, 1% Triton X-100, 0.02 U/µL RNasin, 1× protease inhibitor and 0.1 mg/mL cycloheximide, or 0.2 g of frozen 0–2 h embryos were homogenized in lysis buffer composed of 30 mM Tris-HCl, 100 mM NaCl, 10 mM MgCl_2_, 0.5 mM DTT, 1% Triton X-100, 0.02 U/µL RNasin, 1× protease inhibitor and 0.1 mg/mL cycloheximide. Embryo extracts were incubated for 30 min on ice. Homogenates were cleared by full speed centrifugation for 30 min at 4 °C. For treatment with puromycin, fresh embryos were homogenized in lysis buffer composed of 20 mM Tris-HCl, 140 mM KCl, 0.5 mM DTT, 1% Triton X-100, 0.02 U/µL RNasin, 1× protease inhibitor and 2 mM puromycin. Extracts were incubated for 20 min on ice followed by 20 min at 37 °C and cleared as before. The amount of RNA in the extracts was quantified using Nanodrop. Volumes of extract containing equal amounts of RNA were loaded on top of 10%–50% sucrose gradient containing cycloheximide except for puromycin-treated samples. Gradients were centrifuged for 2 h at 34,000 rpm in a SW41 rotor, with no brake. 1 mL fractions were collected using a ISCO gradient collector. 900 µL of each fraction were used for RNA extraction and 100 µL for protein precipitation. For RNA extraction, 500 pg of luciferase RNA was added to 900 µL of fraction to control RNA extraction. 0.5% SDS and 10 mM EDTA was then added to each fraction, followed by a 5 min incubation. Then, RNAs were prepared using acid phenol-chloroform. One volume of acid phenol-chloroform was added, and the mixture was vortexed and centrifuged at 12,000× *g* for 15 min. The aqueous phase was put in a new tube with 2 µL of glycoblue and 1 volume of isopropanol, incubated for 15 min and centrifuged for 15 min at 12,000× *g*. The RNA pellet was washed twice with 1 volume of 75% ethanol. The RNA pellet was dried for 5 min and resuspended in 20 µL of RNase-free H_2_O. 5 µL of each RNA sample were used for cDNA synthesis using superscript III and random primers. cDNAs were diluted at 1:10 for qPCR reactions that were performed using LightCycler 480 (Roche) and the primers are listed in Supplementary information, Table S3. Data were analyzed using the ΔCp method.^70^ For protein preparation, 400 µL of methanol and 100 µL of chloroform were added to 100 µL of fraction and the mixture was vortexed. The mixture was then added with 300 µL of H_2_O, vortexed and centrifuged at full speed for 5 min. The upper phase was discarded, 435 µL of methanol were added and the mixture was centrifuged at full speed for 5 min. The protein pellet was dried for 1–2 min and resuspended in 100 µL of 2× Laemmli buffer supplemented with 10% β-mercaptoethanol. Proteins were analyzed by western blot; antibodies and dilutions were mouse anti-Aub (4D10, 1:5000),^68^ rabbit anti-PABP (1:1000, gift from A. Vincent), rabbit anti-RpL10Ab (1:5000)^71^ and rabbit anti-RpS3 (1:1000).^71^

### Cloning and recombineering

To produce the *UASp-HA-eIF3d* and *UASp-HA-eIF3d*^*helix11*^ transgenes, eIF3d and eIF3d^helix11^ coding sequences were amplified by PCR from clones provided by S. Rumpf.^49^ PCR fragments were cloned into pENTR by directional Topo cloning (Invitrogen). The generated plasmids were used in gateway cloning to insert the sequences into pPHW (UASp-HA-attR1-ccdB-attR2-SV40 3′UTR) in which an attB (pPHW-attB) site has been inserted. The resulting fragments were then inserted in the *Drosophila* genome by PhiC31 recombination into the attP40 site (BestGene).

To produce FLAG-FFL-Aub, Aub-coding sequence was PCR amplified from the p8161 plasmid^66^ and cloned into pENTR by directional Topo cloning (Invitrogen). The resulting plasmid was used in gateway cloning to insert Aub sequence into pAct-FLAG-Firefly-RfA^72^ (pAFW (DGRC) in which the FFL-coding sequence has been added). To produce HA-RL tagged versions of eIF3b (DGRC, FI08008), eIF3d,^49^ eIF3f (DGRC, LD47792), eIF3k (DGRC, LD03569) and eIF4E (from E. Wahle), the coding sequences were amplified by PCR and cloned into pENTR by directional Topo cloning. The resulting plasmids were used in gateway cloning to insert the coding sequences into pAct-HA-Renilla-RfA^72^ (pAHW (DGRC) in which the RL-coding sequence has been added). For the HA-RL tagged versions of PABP (DNASU, DmCD00772781), eIF3g (DNASU, DmCD00766429), eIF3h (DNASU, DmCD00764259) and eIF4a (DNASU, DmCD00764657), plasmids were directly used in gateway cloning to insert the sequence into pAct-HA-Renilla-RfA. FLAG-FFL-Cherry, HA-RL-Cherry and Sd-RL-HA^72^ were used as negative controls. To produce GST-PABP clones, the coding sequences of five *pAbp* domains, RRM1, RRM2, RRM3, RRM4 and PABC were amplified by PCR from a plasmid provided by E. Wahle. A stop codon (TAA) was added at the end of each domain. The different fragments were cloned into pGEX-4T-1 (Sigma) digested with *Eco*RI and *Xho*I. The plasmids containing HA-Aub(1-482) and HA-Aub(476-866) fragments were generated previously.^13^ The primers used to generate the constructs and the constructs are listed in Supplementary information, Tables S3 and 4, respectively.

### GST pull-down assays

The plasmids containing GST-RRM1, GST-RRM2, GST-RRM3, GST-RRM4 and GST-PABC were introduced in *E. coli* BL21. Protein production was induced by IPTG treatment overnight at 18 °C, or at 37 °C for GST-RRM2. GST-fused proteins were affinity-purified on glutathione-Sepharose 4B beads (GE Healthcare); the beads were incubated overnight at 4 °C in PBT, cOmplete^TM^ EDTA-free Protease Inhibitor Cocktail (Roche) and 5% BSA. HA-Aub proteins were synthesized in vitro using the TnT Coupled reticulocyte lysate system (Promega), and were incubated with immobilized GST fusion proteins in 400 μL binding buffer (50 mM HEPES, pH 7.5, 500 mM NaCl, 0.2 mM EDTA, 1 mM DTT, 0.5% NP-40, cOmplete^TM^ EDTA-free Protease Inhibitor Cocktail (Roche)) containing 0.2 μg/μL RNase A. Incubations were performed for 1 h at 4 °C, followed by 30 min at room temperature. Glutathione-Sepharose beads were then washed four times with binding buffer at room temperature. Recombinant proteins were dissociated from the beads by boiling for 5 min in Laemmli buffer and separated on a SDS-PAGE gel. Western blots were revealed with mouse anti-HA antibody (Covance, MMS-101R) at dilution of 1:1000.

### Quantification and statistical analysis

#### Statistical analysis of mass spectrometric data

Individual LFQ values per detected peptides were first quantile normalized given the experimental condition by using the ProStar (prostar-proteomics.org)^73^ software with the default parameter set. After normalization an imputation step was applied in cases where only one value was missing in each condition group by replacing the missing data by the mean of the observed value for this peptide in their respective experimental condition. Then, each individual experiment was combined into one data matrix. To account for batch effects, ComBat from the R package sva was used. After quality controls, differential expression analysis was done using Reproducibility-Optimized Test Statistic (ROTS)^74^ for each different comparison. *P*-values and FDR were extracted and plotted using self-written R scripts. Significant proteins were annotated using the FlyMine database.^75^

#### Immunofluorescence quantification

Fluorescent images were acquired using a Leica SP8 confocal scanning microscope. Quantification of fluorescent signal was performed using ImageJ tool Measure.

#### Colocalization quantification

Quantification of colocalization in Fig. 2 was performed in 3D using the Imaris software. For colocalization in granules (around nuclei), spots were defined with a minimal size of 0.5 µm and a PSF correction was applied to account for confocal acquisition deformation. Spot colocalization was determined within a radius of 0.25 µm around the center of the spot. For colocalization in foci (between nuclei), spots were defined with a minimal size of 0.2 µm and a PSF correction was applied to account for confocal acquisition deformation. Spot colocalization was determined within a radius of 0.25 µm around the center of the spot. Quantification of colocalization and overlapping signals in Supplementary information, Fig. S3 was performed using ImageJ, with four embryos per staining. Lines were drawn across each GFP-Aub germ granules to obtain the intensity profiles of GFP-Aub and PABP, or GFP-Aub and HA-eIF3d; background signal was subtracted. Each GFP-Aub peak was manually categorized as colocalized, overlapping (single or double) or separated with peaks from the other channel, as depicted in Supplementary information, Fig. S3a, d.

## Supplementary information

Supplementary information, Figure S1

Supplementary information, Figure S2

Supplementary information, Figure S3

Supplementary information, Figure S4

Supplementary information, Figure S5

Supplementary information, Figure S6

Supplementary information, Table S1

Supplementary information, Table S2

Supplementary information, Table S3

Supplementary information, Table S4

## Data Availability

The mass spectrometry proteomics data have been deposited to the ProteomeXchange Consortium via the PRIDE^76^ partner repository with the dataset identifier PXD016399.

## References

[1] Barckmann B, Simonelig M (2013). Control of maternal mRNA stability in germ cells and early embryos. Biochim. Biophys. Acta.

[2] Gavis ER, Lehmann R (1992). Localization of nanos RNA controls embryonic polarity. Cell.

[3] Bergsten SE, Gavis ER (1999). Role for mRNA localization in translational activation but not spatial restriction of nanos RNA. Development.

[4] Trcek T (2015). *Drosophila* germ granules are structured and contain homotypic mRNA clusters. Nat. Commun..

[5] Dahanukar A, Wharton RP (1996). The Nanos gradient in *Drosophila* embryos is generated by translational regulation. Genes Dev..

[6] Gavis ER, Lehmann R (1994). Translational regulation of nanos by RNA localization. Nature.

[7] Dahanukar A, Walker JA, Wharton RP (1999). Smaug, a novel RNA-binding protein that operates a translational switch in *Drosophila*. Mol. Cell.

[8] Smibert CA, Wilson JE, Kerr K, Macdonald PM (1996). Smaug protein represses translation of unlocalized nanos mRNA in the *Drosophila* embryo. Genes Dev..

[9] Zaessinger S, Busseau I, Simonelig M (2006). Oskar allows nanos mRNA translation in *Drosophila* embryos by preventing its deadenylation by Smaug/CCR4. Development.

[10] Ephrussi A, Lehmann R (1992). Induction of germ cell formation by oskar. Nature.

[11] Jeske M, Moritz B, Anders A, Wahle E (2011). Smaug assembles an ATP-dependent stable complex repressing nanos mRNA translation at multiple levels. EMBO J.

[12] Barckmann B (2015). Aubergine iCLIP reveals piRNA-dependent decay of mRNAs involved in germ cell development in the early embryo. Cell Rep..

[13] Dufourt J (2017). piRNAs and Aubergine cooperate with Wispy poly(A) polymerase to stabilize mRNAs in the germ plasm. Nat. Commun..

[14] Czech B (2018). piRNA-guided genome defense: from biogenesis to silencing. Annu. Rev. Genet..

[15] Huang X, Fejes Toth K, Aravin A (2017). piRNA biogenesis in *Drosophila**melanogaster*. Trends Genet..

[16] Rojas-Rios P, Simonelig M (2018). piRNAs and PIWI proteins: regulators of gene expression in development and stem cells. Development.

[17] Mani SR, Megosh H, Lin H (2014). PIWI proteins are essential for early *Drosophila* embryogenesis. Dev. Biol..

[18] Rouget C (2010). Maternal mRNA deadenylation and decay by the piRNA pathway in the early *Drosophila* embryo. Nature.

[19] Vourekas A, Alexiou P, Vrettos N, Maragkakis M, Mourelatos Z (2016). Sequence-dependent but not sequence-specific piRNA adhesion traps mRNAs to the germ plasm. Nature.

[20] Goh WS (2015). piRNA-directed cleavage of meiotic transcripts regulates spermatogenesis. Genes Dev..

[21] Gou LT (2014). Pachytene piRNAs instruct massive mRNA elimination during late spermiogenesis. Cell Res..

[22] Kiuchi T (2014). A single female-specific piRNA is the primary determiner of sex in the silkworm. Nature.

[23] Watanabe T, Cheng EC, Zhong M, Lin H (2015). Retrotransposons and pseudogenes regulate mRNAs and lncRNAs via the piRNA pathway in the germline. Genome Res..

[24] Zhang P (2015). MIWI and piRNA-mediated cleavage of messenger RNAs in mouse testes. Cell Res..

[25] Riechmann V, Gutierrez GJ, Filardo P, Nebreda AR, Ephrussi A (2002). Par-1 regulates stability of the posterior determinant Oskar by phosphorylation. Nat. Cell Biol..

[26] Markussen FH, Michon AM, Breitwieser W, Ephrussi A (1995). Translational control of oskar generates short OSK, the isoform that induces pole plasma assembly. Development.

[27] Malone CD (2009). Specialized piRNA pathways act in germline and somatic tissues of the *Drosophila* ovary. Cell.

[28] Sienski G (2015). Silencio/CG9754 connects the Piwi-piRNA complex to the cellular heterochromatin machinery. Genes Dev..

[29] Yu Y (2015). Panoramix enforces piRNA-dependent cotranscriptional silencing. Science.

[30] Khurana JS, Xu J, Weng Z, Theurkauf WE (2010). Distinct functions for the *Drosophila* piRNA pathway in genome maintenance and telomere protection. PLoS Genet.

[31] Little SC, Sinsimer KS, Lee JJ, Wieschaus EF, Gavis ER (2015). Independent and coordinate trafficking of single *Drosophila* germ plasm mRNAs. Nat. Cell Biol..

[32] Thomson T, Liu N, Arkov A, Lehmann R, Lasko P (2008). Isolation of new polar granule components in *Drosophila* reveals P body and ER associated proteins. Mech. Dev..

[33] Kirino Y (2010). Arginine methylation of Aubergine mediates Tudor binding and germ plasm localization. RNA.

[34] Nishida KM (2009). Functional involvement of Tudor and dPRMT5 in the piRNA processing pathway in *Drosophila* germlines. EMBO J..

[35] Gotze M (2017). Translational repression of the *Drosophila* nanos mRNA involves the RNA helicase Belle and RNA coating by Me31B and Trailer hitch. RNA.

[36] Kirino Y (2009). Arginine methylation of Piwi proteins catalysed by dPRMT5 is required for Ago3 and Aub stability. Nat. Cell Biol..

[37] Trepte P (2015). DULIP: a dual luminescence-based co-immunoprecipitation assay for interactome mapping in mammalian cells. J. Mol. Biol..

[38] Brook M, Smith JW, Gray NK (2009). The DAZL and PABP families: RNA-binding proteins with interrelated roles in translational control in oocytes. Reproduction.

[39] Valasek LS (2017). Embraced by eIF3: structural and functional insights into the roles of eIF3 across the translation cycle. Nucleic Acids Res..

[40] Lee AS, Kranzusch PJ, Cate JH (2015). eIF3 targets cell-proliferation messenger RNAs for translational activation or repression. Nature.

[41] Lee AS, Kranzusch PJ, Doudna JA, Cate JH (2016). eIF3d is an mRNA cap-binding protein that is required for specialized translation initiation. Nature.

[42] Beilharz TH, Preiss T (2004). Translational profiling: the genome-wide measure of the nascent proteome. Brief Funct. Genom. Proteom..

[43] Benoit B (2009). An essential role for the RNA-binding protein Smaug during the *Drosophila* maternal-to-zygotic transition. Development.

[44] Tadros W (2007). SMAUG is a major regulator of maternal mRNA destabilization in *Drosophila* and its translation is activated by the PAN GU kinase. Dev. Cell.

[45] Kronja I (2014). Widespread changes in the posttranscriptional landscape at the *Drosophila* oocyte-to-embryo transition. Cell Rep..

[46] Grivna ST, Pyhtila B, Lin H (2006). MIWI associates with translational machinery and PIWI-interacting RNAs (piRNAs) in regulating spermatogenesis. Proc. Natl. Acad. Sci. USA.

[47] Unhavaithaya Y (2009). MILI, a PIWI-interacting RNA-binding protein, is required for germ line stem cell self-renewal and appears to positively regulate translation. J. Biol. Chem..

[48] Szostak E (2018). Hrp48 and eIF3d contribute to msl-2 mRNA translational repression. Nucleic Acids Res..

[49] Rode S (2018). Differential requirement for translation initiation factor pathways during ecdysone-dependent neuronal remodeling in *Drosophila*. Cell Rep..

[50] Meyer KD (2015). 5’ UTR m(6)A promotes cap-independent translation. Cell.

[51] Thakor N (2017). Cellular mRNA recruits the ribosome via eIF3-PABP bridge to initiate internal translation. RNA Biol.

[52] Nelson MR, Leidal AM, Smibert CA (2004). *Drosophila* cup is an eIF4E-binding protein that functions in Smaug-mediated translational repression. EMBO J..

[53] Igreja C, Izaurralde E (2011). CUP promotes deadenylation and inhibits decapping of mRNA targets. Genes Dev..

[54] Chen Y (2014). A DDX6-CNOT1 complex and W-binding pockets in CNOT9 reveal direct links between miRNA target recognition and silencing. Mol. Cell.

[55] Mathys H (2014). Structural and biochemical insights to the role of the CCR4-NOT complex and DDX6 ATPase in microRNA repression. Mol. Cell.

[56] Dodson AE, Kennedy S (2019). Germ granules coordinate RNA-based epigenetic inheritance pathways. Dev. Cell.

[57] Ouyang JPT (2019). P granules protect RNA interference genes from silencing by piRNAs. Dev. Cell.

[58] Rangan P (2009). Temporal and spatial control of germ-plasm RNAs. Curr. Biol..

[59] Weil TT (2012). *Drosophila* patterning is established by differential association of mRNAs with P bodies. Nat. Cell Biol..

[60] Dai P (2019). A translation-activating function of MIWI/piRNA during mouse spermiogenesis. Cell.

[61] Schupbach T, Wieschaus E (1991). Female sterile mutations on the second chromosome of *Drosophila**melanogaster*. II. Mutations blocking oogenesis or altering egg morphology. Genetics.

[62] Rorth P (1998). Gal4 in the *Drosophila* female germline. Mech. Dev..

[63] Tomari Y (2004). RISC assembly defects in the *Drosophila* RNAi mutant armitage. Cell.

[64] Cook HA, Koppetsch BS, Wu J, Theurkauf WE (2004). The *Drosophila* SDE3 homolog armitage is required for oskar mRNA silencing and embryonic axis specification. Cell.

[65] Forrest KM, Clark IE, Jain RA, Gavis ER (2004). Temporal complexity within a translational control element in the nanos mRNA. Development.

[66] Harris AN, Macdonald PM (2001). Aubergine encodes a *Drosophila* polar granule component required for pole cell formation and related to eIF2C. Development.

[67] Chartier A (2015). Mitochondrial dysfunction reveals the role of mRNA poly(A) tail regulation in oculopharyngeal muscular dystrophy pathogenesis. PLoS Genet..

[68] Gunawardane LS (2007). A slicer-mediated mechanism for repeat-associated siRNA 5’ end formation in *Drosophila*. Science.

[69] Cox J, Mann M (2008). MaxQuant enables high peptide identification rates, individualized p.p.b.-range mass accuracies and proteome-wide protein quantification. Nat. Biotechnol..

[70] Panda AC, Martindale JL, Gorospe M (2017). Polysome fractionation to analyze mRNA distribution profiles. Bio-Protocol.

[71] Antic S, Wolfinger MT, Skucha A, Hosiner S, Dorner S (2015). General and microRNA-mediated mRNA degradation occurs on ribosome complexes in *Drosophila* cells. Mol. Cell Biol..

[72] Srivastava, D., de Toledo, M., Manchon, L., Tazi, J. & Juge, F. Modulation of Yorkie activity by alternative splicing is required for developmental stability. 10.1101/2019.12.19.882779 (2019). 10.15252/embj.2020104895 PMC7849169 33320356

[73] Wieczorek S (2017). DAPAR & ProStaR: software to perform statistical analyses in quantitative discovery proteomics. Bioinformatics.

[74] Suomi, T., Seyednasrollah, F., Jaakkola, M. K., Faux, T. & Elo, L. L. ROTS: an R package for reproducibility-optimized statistical testing. *PLoS Comput. Biol.* **13**, e1005562 (2017). 10.1371/journal.pcbi.1005562 PMC5470739 28542205

[75] Lyne R (2007). FlyMine: an integrated database for *Drosophila* and *Anopheles* genomics. Genome Biol.

[76] Perez-Riverol Y (2019). The PRIDE database and related tools and resources in 2019: improving support for quantification data. Nucleic Acids Res..

