# Supplementary material for: The PIWI protein Aubergine recruits eIF3 to activate translation in the germ plasm
Source: Cell Res. 2020 Mar 4;30(5):421–35. doi: 10.1038/s41422-020-0294-9 (PMC7196074; doi:10.1038/s41422-020-0294-9)
Supplement: Supplementary file 1 — Supplementary information, Figure S1 [file 41422_2020_294_MOESM1_ESM.pdf]

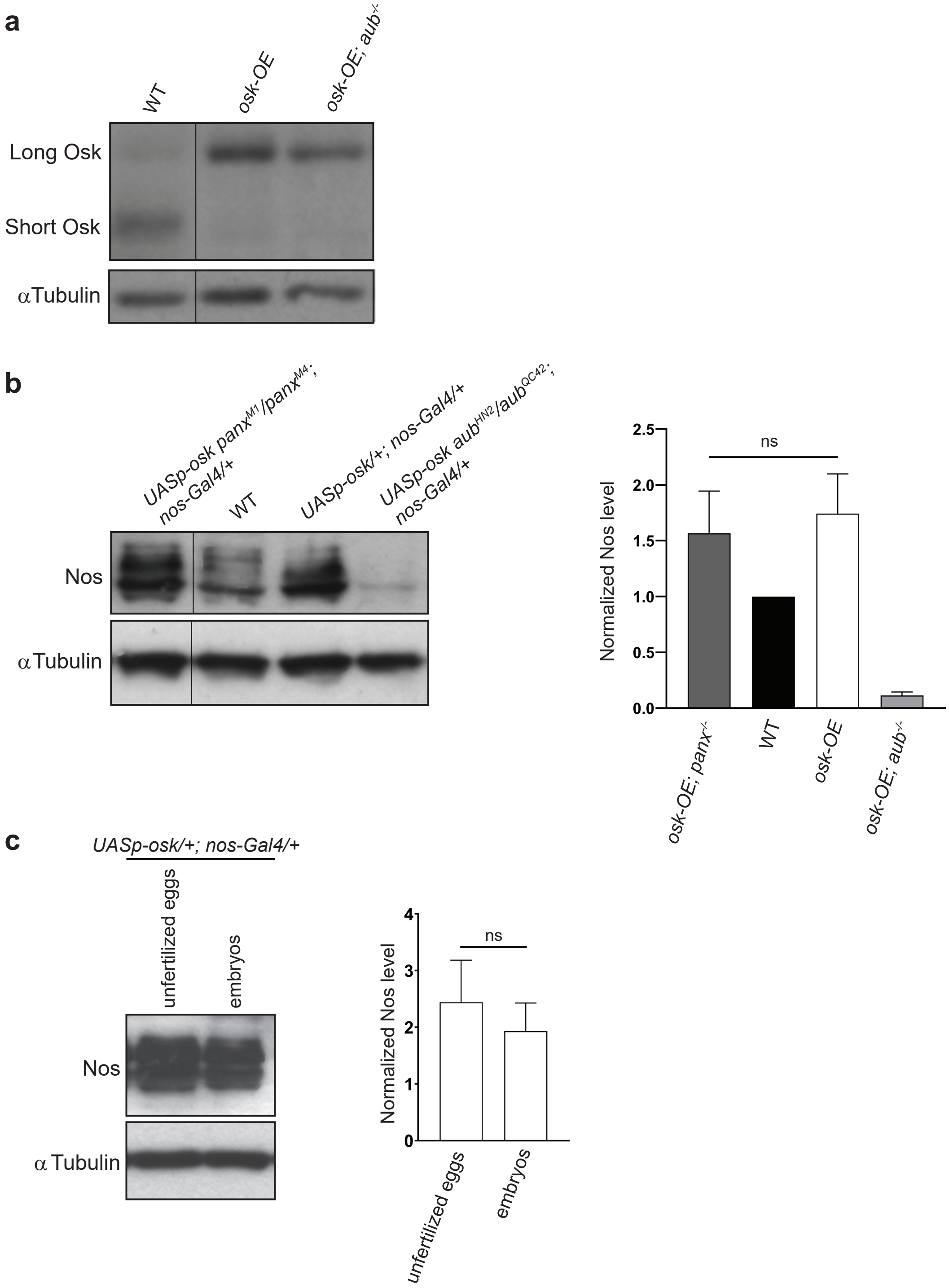
a
WT
osk-OE
osk-OE; aub-/-
Long Osk
Short Osk
αTubulin
b
UASp-osk panxM1/panxM4; nos-Gal4/+
WT
UASp-osk/+; nos-Gal4/+
UASp-osk aubHN2/aubQC42; nos-Gal4/+
Nos
αTubulin
ns
Normalized Nos level
2.5
2.0
1.5
1.0
0.5
0.0
osk-OE; panx-/-
WT
osk-OE
osk-OE; aub-/-
c
UASp-osk/+; nos-Gal4/+
unfertilized eggs
embryos
Nos
α Tubulin
ns
Normalized Nos level
4
3
2
1
0
unfertilized eggs
embryos

Figure S1

Fig. S1 ***nos*** **mRNA translation is independent of Panx and development. a** Western blots of wild-type (WT), *osk-OE* and *osk-OE; aub*$^{-/-}$ embryos revealed with anti-Osk showing that Long Osk is overexpressed from the *UASp-osk* transgene. α-Tubulin was used as a loading control. **b** Western blots of WT embryos and embryos overexpressing *osk* either in a WT, *aub* mutant or *panx* mutant background revealed with anti-Nos, showing that Nos levels were not affected in the *panx* mutant background. α-Tubulin was used as a loading control. Quantification was performed using the ImageJ software with 4 biological replicates. Error bars represent SEM. ns: not significant, using the unpaired Student's *t*-test. **c** Western blots of *osk-OE* embryos and unfertilized eggs, showing that Nos levels did not depend on embryonic development. α-Tubulin was used as a loading control. Quantification was performed using the ImageJ software with 4 biological replicates. Error bars represent SEM. ns: not significant, using the unpaired Student's *t*-test.
