# Supplementary material for: The PIWI protein Aubergine recruits eIF3 to activate translation in the germ plasm
Source: Cell Res. 2020 Mar 4;30(5):421–35. doi: 10.1038/s41422-020-0294-9 (PMC7196074; doi:10.1038/s41422-020-0294-9)
Supplement: Supplementary file 2 — Supplementary information, Figure S2 [file 41422_2020_294_MOESM2_ESM.pdf]

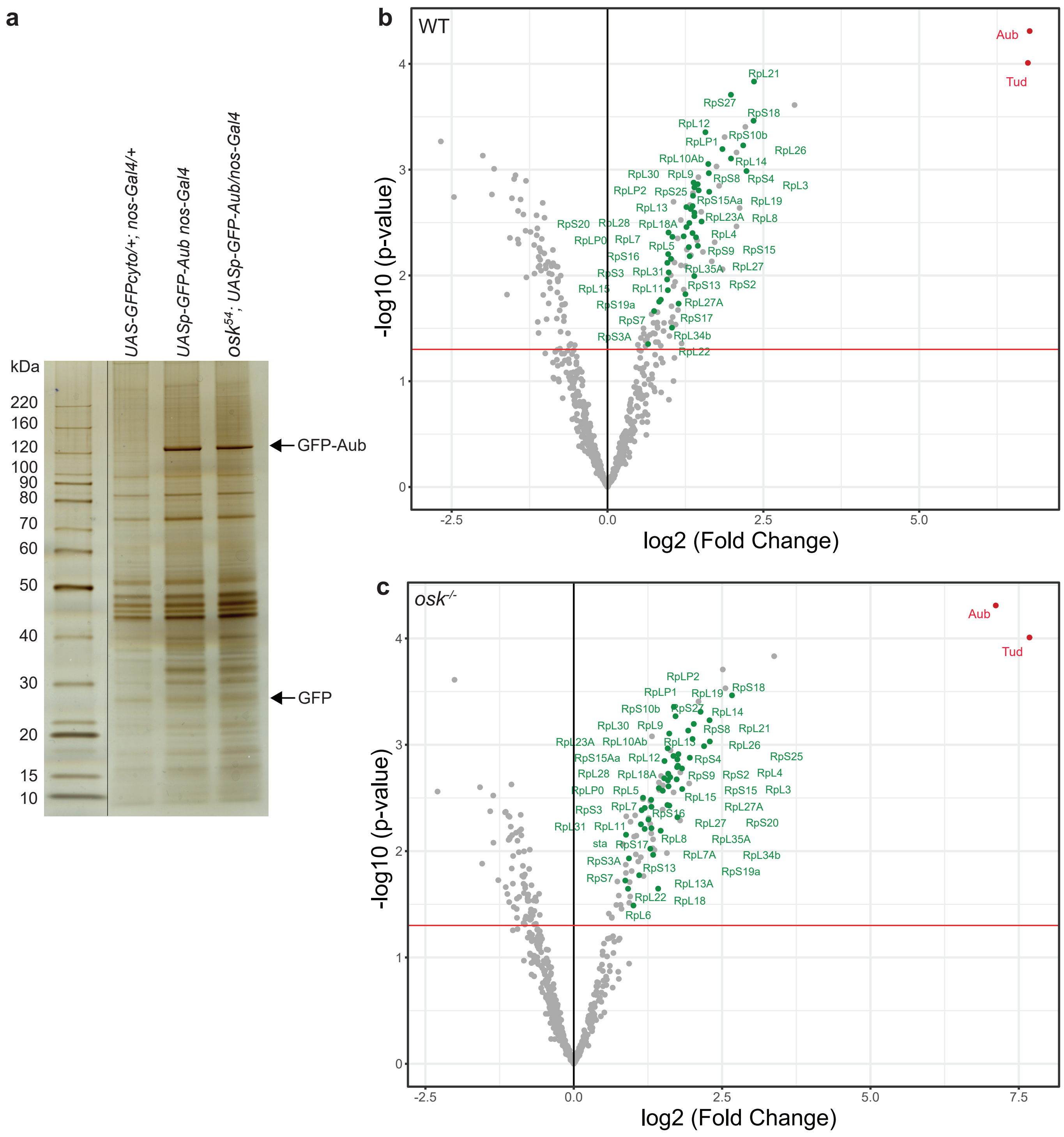
a
UAS-GFPcyto/+; nos-Gal4/+
UASp-GFP-Aub nos-Gal4
osk54; UASp-GFP-Aub/nos-Gal4
kDa
220
160
120
100
90
80
70
60
50
40
30
20
15
10
GFP-Aub
GFP
b
WT
Aub
Tud
log2 (Fold Change)
-log10 (p-value)
c
osk-/-
Aub
Tud
log2 (Fold Change)
-log10 (p-value)

Figure S2

Fig. S2 Ribosomal proteins are associated with Aub. a Silver stained gel showing protein extracts used for mass spectrometry following GFP IP. **b, c** Volcano plots showing the mass spectrometry analysis of GFP-Aub IP from 0-2 hour-embryos. Embryos expressing cytoplasmic GFP were used as control. (**b**) *UASp-GFP-Aub nos-Gal4* embryos; (**c**) *osk*^{54}*; UASp-GFP-Aub/nos-Gal4* embryos. The analysis was based on four biological replicates. The red line indicates the significance threshold (*P* = 0.05). Ribosomal proteins are indicated in green.
