# Supplementary material for: The PIWI protein Aubergine recruits eIF3 to activate translation in the germ plasm
Source: Cell Res. 2020 Mar 4;30(5):421–35. doi: 10.1038/s41422-020-0294-9 (PMC7196074; doi:10.1038/s41422-020-0294-9)
Supplement: Supplementary file 3 — Supplementary information, Figure S3 [file 41422_2020_294_MOESM3_ESM.pdf]

a

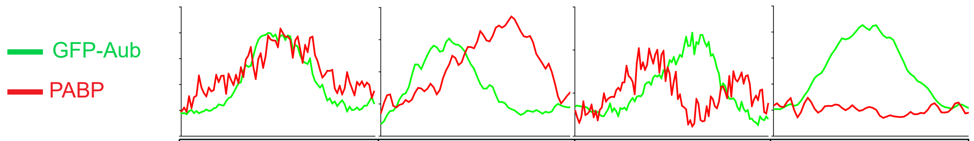

	% colocalized foci	% single overlaping foci	% double overlaping foci	% separated foci
Aub in PABP (n=134)	31.2% ± 2.7	25.3% ± 3.3	22.9% ± 3.5	20.6% ± 5.5

b

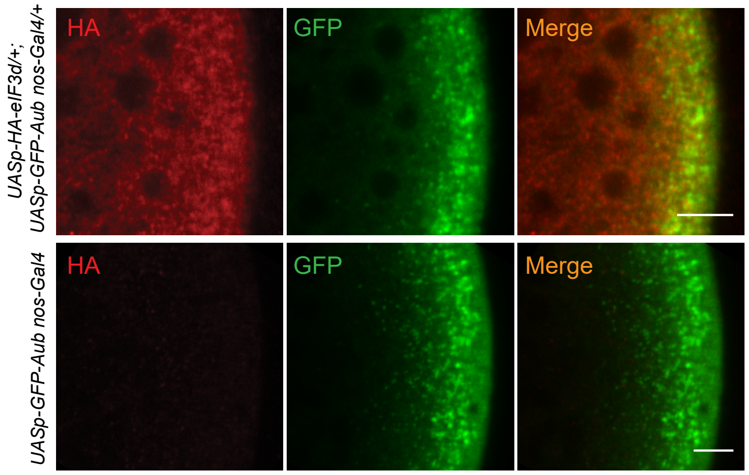

c

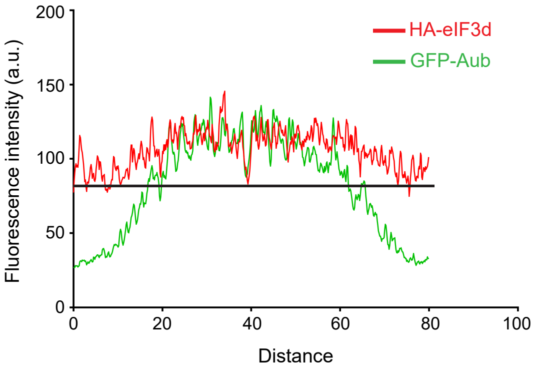

d

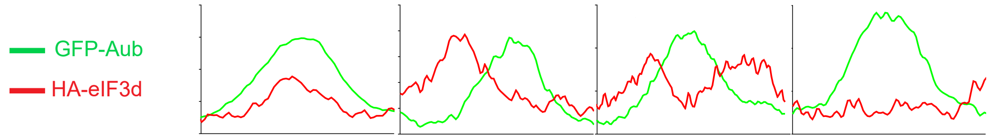

	% colocalized foci	% single overlaping foci	% double overlaping foci	% separated foci
Aub in HA-eIF3d (n=143)	34.3% ± 2.4	31.2 ± 0.9	17.7% ± 2.1	16.8% ± 0.3

Figure S3

Fig. S3 PABP and eIF3d foci colocalize or overlap with germ granules. a Quantification of colocalization and overlap between Aub-containing germ granules and PABP foci. Representative graphs for each category (colocalization; single overlap; double overlap; separated foci) are shown. Quantification was performed using the ImageJ software. **b** Immunostaining of embryos with anti-GFP (green) to visualize Aub and anti-HA (red), showing the specificity of anti-HA antibody that did not recognize any protein in embryos lacking the *UASp-HA-eIF3d* transgene. Posterior of embryos are shown. Scale bars: 5 μm. **c** Graph showing the slight accumulation of eIF3d at the posterior pole of embryos. A line was drawn along the posterior cortex of the embryo shown in Fig. 4i and the fluorescence intensity was quantified using the ImageJ software. **d** Quantification of colocalization and overlap between Aub-containing germ granules and HA-eIF3d foci. Representative graphs for each category (colocalization; single overlap; double overlap; separated foci) are shown. Quantification was performed using the ImageJ software.
