# Supplementary material for: The PIWI protein Aubergine recruits eIF3 to activate translation in the germ plasm
Source: Cell Res. 2020 Mar 4;30(5):421–35. doi: 10.1038/s41422-020-0294-9 (PMC7196074; doi:10.1038/s41422-020-0294-9)
Supplement: Supplementary file 4 — Supplementary information, Figure S4 [file 41422_2020_294_MOESM4_ESM.pdf]

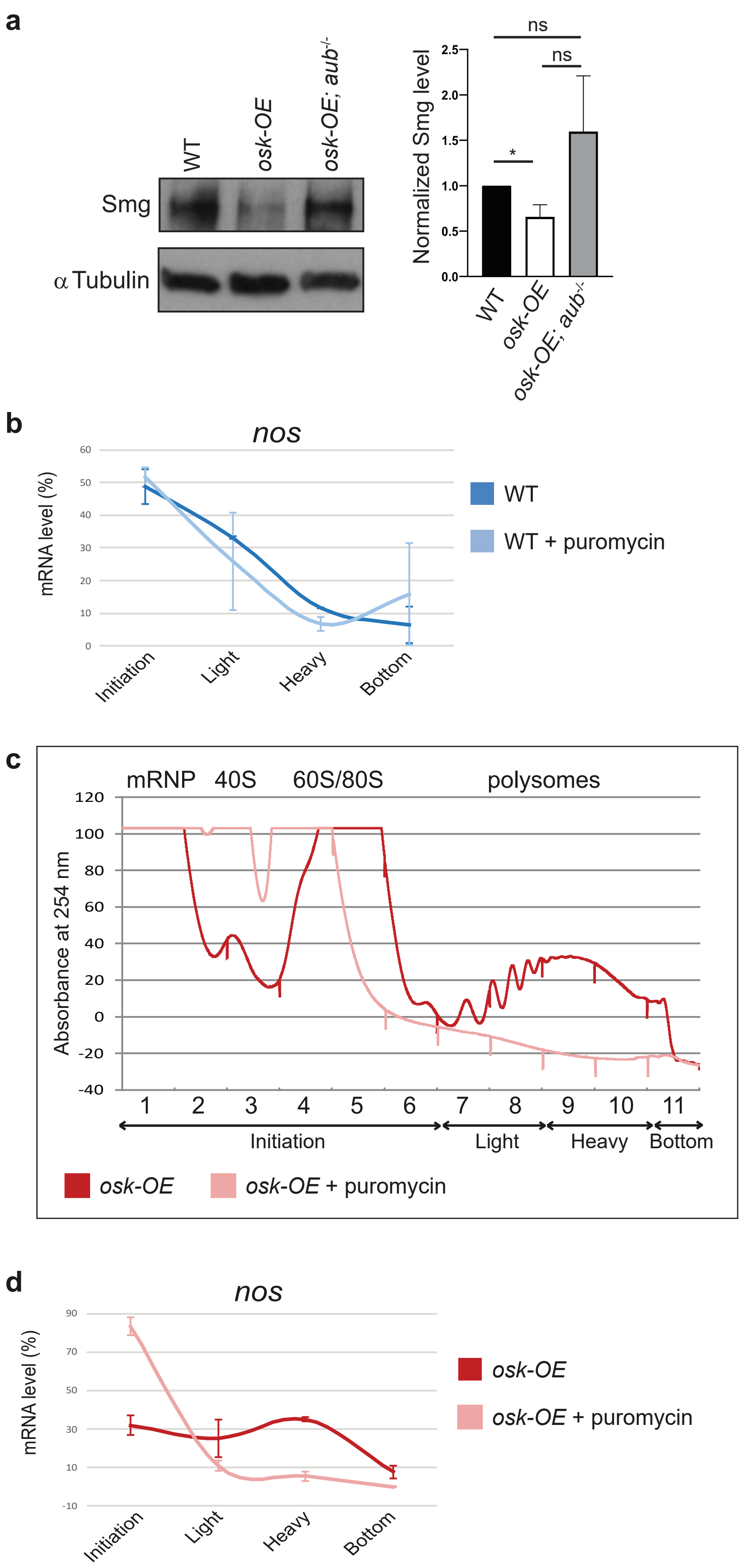

a
WT
osk-OE
osk-OE; aub-/-
Smg
α Tubulin
ns
ns
*
Normalized Smg level
2.5
2.0
1.5
1.0
0.5
0.0
b
nos
mRNA level (%)
60
50
40
30
20
10
0
Initiation
Light
Heavy
Bottom
WT
WT + puromycin
c
mRNP
40S
60S/80S
polysomes
Absorbance at 254 nm
120
100
80
60
40
20
0
-20
-40
1
2
3
4
5
6
7
8
9
10
11
Initiation
Light
Heavy
Bottom
osk-OE
osk-OE + puromycin
d
nos
mRNA level (%)
90
70
50
30
10
-10
Initiation
Light
Heavy
Bottom
osk-OE
osk-OE + puromycin

Figure S4

Fig. S4 *nos* mRNA profiling through polysome gradients. a Western blots of wild-type (WT), *osk-OE* and *osk-OE; aub*$^{-/-}$ embryos revealed with anti-Smg showing that Smg levels did not decrease in *osk-OE; aub*$^{-/-}$ embryos. α-Tubulin was used as a loading control. Quantification was performed using the ImageJ software with 4 biological replicates. Error bars represent SEM. *$P < 0.05$, ns: not significant, using the unpaired Student's *t*-test. **b** Quantification of *nos* mRNA using RT-qPCR in the different fractions of the gradients for WT embryos in the absence (dark blue) or the presence (light blue) of puromycin. mRNA levels are indicated in percentage of total mRNA in all the fractions. Mean of two biological replicates, quantified in triplicate. Error bars represent SEM. **c** Profile of absorbance at 254 nm for 0-2 hour *osk-OE* embryos treated (pink), or not (red) with puromycin, fractionated into 10%-50 % sucrose gradients. **d** Quantification of *nos* mRNA using RT-qPCR in the different fractions of the gradients for *osk-OE* embryos in the absence (red) or the presence (pink) of puromycin. mRNA levels are indicated in percentage of total mRNA in all the fractions. Mean of two biological replicates, quantified in triplicate. Error bars represent SEM.
