# Supplementary material for: The PIWI protein Aubergine recruits eIF3 to activate translation in the germ plasm
Source: Cell Res. 2020 Mar 4;30(5):421–35. doi: 10.1038/s41422-020-0294-9 (PMC7196074; doi:10.1038/s41422-020-0294-9)
Supplement: Supplementary file 5 — Supplementary information, Figure S5 [file 41422_2020_294_MOESM5_ESM.pdf]

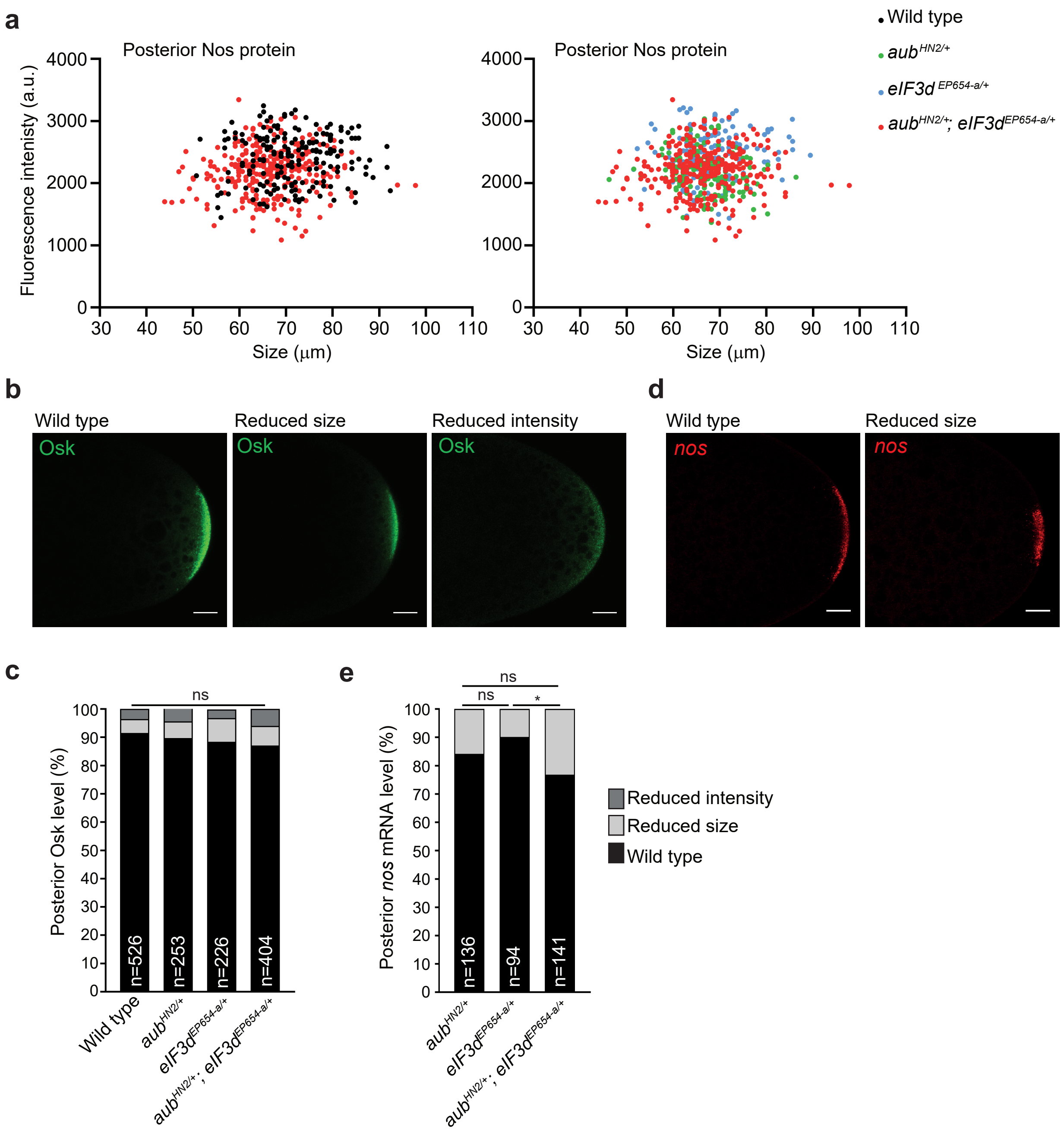
a
Posterior Nos protein
Posterior Nos protein
Fluorescence intenisty (a.u.)
Size (μm)
Size (μm)
Wild type
aubHN2/+
eIF3d EP654-a/+
aubHN2/+; eIF3dEP654-a/+
b
Wild type
Reduced size
Reduced intensity
Osk
Osk
Osk
d
Wild type
Reduced size
nos
nos
c
ns
Posterior Osk level (%)
n=526
n=253
n=226
n=404
Wild type
aubHN2/+
eIF3dEP654-a/+
aubHN2/+; eIF3dEP654-a/+
e
ns
ns
*
Posterior nos mRNA level (%)
n=136
n=94
n=141
aubHN2/+
eIF3dEP654-a/+
aubHN2/+; eIF3dEP654-a/+
Reduced intensity
Reduced size
Wild type

Figure S5

Fig. S5 Aub-eIF3d interaction does not regulate Osk posterior accumulation. a Scatter plots of size and fluorescence intensity of Nos posterior immunostaining for each embryo of the indicated genotype, showing embryos with reduced size and fluorescence intensity in the double heterozygous mutant compared to wild type or single mutants, but no general decrease in fluorescence intensity among all double heterozygous mutant embryos. Left panel: wild type and double *aub*, *eIF3d* heterozygous mutant. Right panel: single and double *aub*, *eIF3d* heterozygous mutants. **b, c** Immunostaining of wild-type, single and double *aub*, *eIF3d* heterozygous mutant embryos with anti-Osk antibody. Posterior of embryos showing the three types of staining: wild type, reduced size or reduced intensity (**b**). For each genotype, the percentage of embryos with each staining category was recorded (**c**). ns: not significant, using the χ^2 test. **d, e** *nos* smFISH of single and double *aub*, *eIF3d* heterozygous mutant embryos. Posterior of embryos showing the two types of staining: wild type and reduced size (**d**). For each genotype, the percentage of embryos with each staining category was recorded (**e**). *$P < 0.05$, ns: not significant, using the χ^2 test. Scale bars: 20 μm in **b** and **d**.
