# Supplementary material for: The PIWI protein Aubergine recruits eIF3 to activate translation in the germ plasm
Source: Cell Res. 2020 Mar 4;30(5):421–35. doi: 10.1038/s41422-020-0294-9 (PMC7196074; doi:10.1038/s41422-020-0294-9)
Supplement: Supplementary file 6 — Supplementary information, Figure S6 [file 41422_2020_294_MOESM6_ESM.pdf]

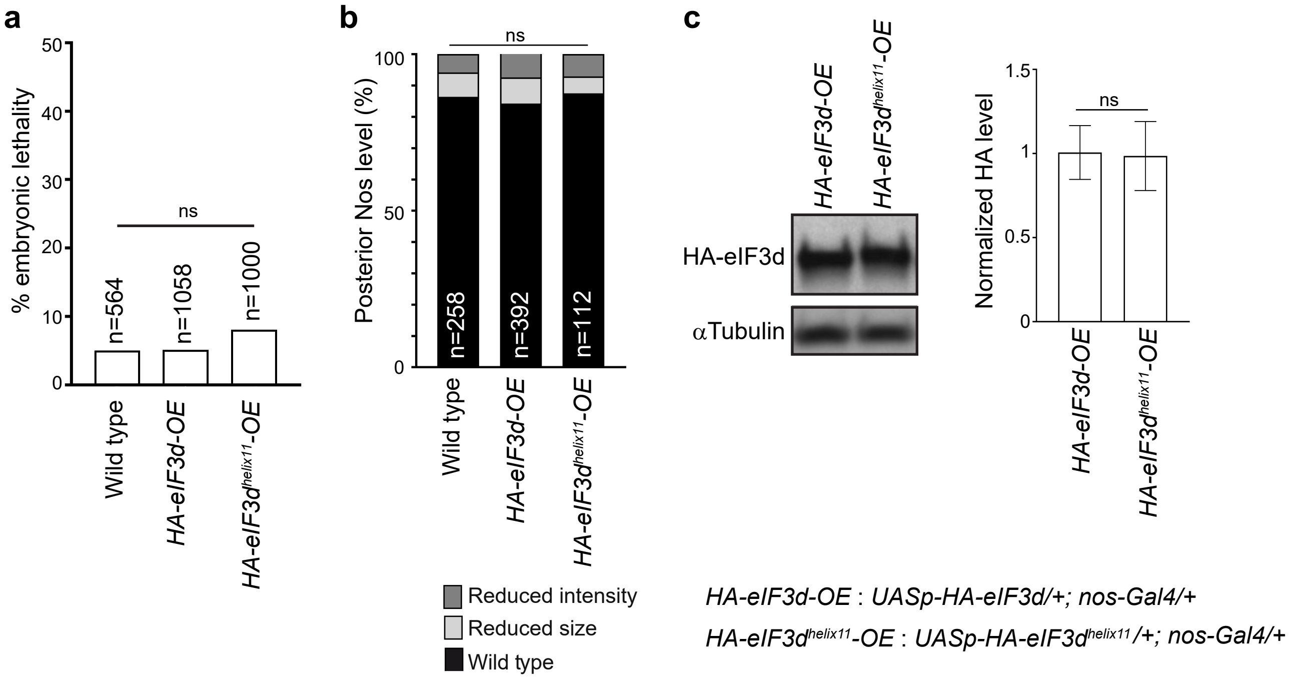
a
% embryonic lethality
ns
n=564
n=1058
n=1000
Wild type
HA-eIF3d-OE
HA-eIF3d^helix11-OE
b
Posterior Nos level (%)
ns
n=258
n=392
n=112
Wild type
HA-eIF3d-OE
HA-eIF3d^helix11-OE
Reduced intensity
Reduced size
Wild type
c
HA-eIF3d-OE
HA-eIF3d^helix11-OE
HA-eIF3d
αTubulin
Normalized HA level
ns
HA-eIF3d-OE : UASp-HA-eIF3d/+; nos-Gal4/+
HA-eIF3d^helix11-OE : UASp-HA-eIF3d^helix11/+; nos-Gal4/+

Figure S6

Fig. S6 *eIF3d*^{helix11} does not act as a negative dominant mutant in the embryo. a Percentage of lethality of embryos overexpressing HA-eIF3d or HA-eIF3d^{helix11}. The genotypes are indicated. ns: not significant, using the χ^2 test. **b** Quantification of immunostaining with anti-Nos antibody of embryos overexpressing HA-eIF3d or HAeIF3d^{helix11}. The three types of staining, wild type, reduced size or reduced intensity were as in Fig. 6b. For each genotype, the percentage of embryos with each staining category was recorded. ns: not significant, using the χ^2 test. **c** Western blot of embryos overexpressing HA-eIF3d or HA-eIF3d^{helix11} revealed with anti-HA, showing that the level of overexpression is similar for eIF3d and eIF3d^{helix11}. α-Tubulin was used as a loading control. Quantification was performed using the ImageJ software with 3 biological replicates. Error bars represent SEM. ns: not significant, using the unpaired Student's *t*-test.
