# Supplementary material for: The PIWI protein Aubergine recruits eIF3 to activate translation in the germ plasm
Source: Cell Res. 2020 Mar 4;30(5):421–35. doi: 10.1038/s41422-020-0294-9 (PMC7196074; doi:10.1038/s41422-020-0294-9)
Supplement: Supplementary file 8 — Supplementary information, Table S2 [file 41422_2020_294_MOESM8_ESM.pdf]

Table S2 Stellaris anti-*nos* probes

ggtaaagctacgcgccaact
acggcaattccaggaatttt
acttgaaacaactgcgaagc
aaaatcgtatgtcccttaga
aaatcgtgacgcagaggcaa
aactaaactcgcttttgggt
ttcgcgatacttctttatct
gcacagtttattcaactgaa
caaatcctcacccaaaaacc
ttatcgcgcactctactttc
aaatccgggtcgaaagttac
taaacgctgcaaaagctgcc
gctttgatcggaatgcgtat
ccaggcgctatttaaacgtt
gcacgggataacgctctaaa
agtgatcgttcgttgtctat
gtttccctttcacagaaaca
gccacgacgattgaacaagt
tccattcatcaactttcgga
aaatgaaggcgaccagttgc
cgaaatttttcggccgcaag
ttcaaagtgttcctttttca
tgatacgattgacagttcga
cttgctatttcctttagcaa
acaatgaatgcgtagccgac
tactcttcgcttatctatca
gcggttgtttcatgtgtgaa
cgagccattgaatttttcat
aaccatttctttatttggca
tccaagttgctgcggaacat
tcctctggcgtgaaaagcag
tgcaggcccagaatgttgag
ccactggtatccaaatacat
gtaatgggcggactcaaagt
tcggccagaaaagggaagtg
cataaggagcgaattggcgg
caagtggtagtggtactgtc
ttgctggtgactcgcactag
aaggatcgcgcaatctcgtc
cgtcacctgcgcaaagattt
catagccattggtcgcgaac
taggacatgcgaccgagatc
cattaagttgccgccattgg
agtgggtggcgagtggaatg
cacacgttgttcagatgctc
ggctggtatatacgacatgt
ctgcaaacccattgtattgg
cgagattggtggacacagtg
