# Supplementary material for: The PIWI protein Aubergine recruits eIF3 to activate translation in the germ plasm
Source: Cell Res. 2020 Mar 4;30(5):421–35. doi: 10.1038/s41422-020-0294-9 (PMC7196074; doi:10.1038/s41422-020-0294-9)
Supplement: Supplementary file 9 — Supplementary information, Table S3 [file 41422_2020_294_MOESM9_ESM.pdf]

Table S3 Primers used in this study

Forward primer luciferase (qPCR)	AGAGATACGCCCTGGTTCCT
Reverse primer luciferase (qPCR)	ATAAATAACGCGCCCAACAC
Forward primer *nos* (qPCR)	CGGAGCTTCCAATTCCAGTAAC
Reverse primer *nos* (qPCR)	AGTTATCTCGCACTGAGTGGCT
Forward primer *smg* (qPCR)	TCCCAAAATGCATCACGTC
Reverse primer *smg* (qPCR)	TCGCATTTGCGAGTCTTACC
Forward primer *mRpL43* (qPCR)	CGGGATTGTGGTCTATGTGA
Reverse primer *mRpL43* (qPCR)	GAGCTGCCGTTTTGAGTCTT
Forward primer *RpL32* (qPCR)	CTTCATCCGCCACCAGTC
Reverse primer *RpL32* (qPCR)	CGACGCACTCTGTTGTCG
Forward primer *eIF3d* (PCR for TOPO cloning)	CACCATGAGCGAGACCATAAACAC
Reverse primer *eIF3d* (PCR for TOPO cloning)	AATCTTGTTGTGCGCGTAGTTGTA
Forward primer *aub* (PCR for TOPO cloning)	CACCATGAATTTACCACCAAACCCTGTAA
Reverse primer *aub* (PCR for TOPO cloning)	TTACAAAAAGTACAATTGATTCTGCAGTCCTGC
Forward primer *eIF3b* (PCR for TOPO cloning)	CACCATGGCCAAGAAGAAAAGCGA
Reverse primer *eIF3b* (PCR for TOPO cloning)	CTCGAGCAGAGTGACTTCTTCCTT
Forward primer *eIF3f* (PCR for TOPO cloning)	CACCATGTCGGCTCTCAATC
Reverse primer *eIF3f* (PCR for TOPO cloning)	CTAGGCGGTGGGCAGGAAG
Forward primer *eIF3k* (PCR for TOPO cloning)	CACCATGTCGCACCTCGTGA
Reverse primer *eIF3k* (PCR for TOPO cloning)	CAGGCACTGGGCCATCAGGG
Forward primer *eIF4E* (PCR for TOPO cloning)	CACCATGCAGAGCGACTTTCACAGA
Reverse primer *eIF4E* (PCR for TOPO cloning)	CAAAGTGTAGATCGATTTCACGTTGG
Forward primer *pAbp* (PCR RRM1 domain for cloning)	ACCGAATTCATGGCTTCTCTATAC
Reverse primer *pAbp* (PCR RRM1 domain for cloning)	ACCCTCGAGTTACACACCTGAGCG
Forward primer *pAbp* (PCR RRM2 domain for cloning)	ACCGAATTCGATCCTTCTCTTCGT
Reverse primer *pAbp* (PCR RRM2 domain for cloning)	ACCCTCGAGTTACTTAGCCTTCTC
Forward primer *pAbp* (PCR RRM3 domain for cloning)	ACCGAATTCCGCAAGGAGCGGAG
Reverse primer *pAbp* (PCR RRM3 domain for cloning)	ACCCTCGAGTTACACAGACTCGTG
Forward primer *pAbp* (PCR RRM4 domain for cloning)	ACCGAATTCCAGAAGAAGGCCGAA
Reverse primer *pAbp* (PCR RRM4 domain for cloning)	ACCCTCGAGTTACTCCTCCTTCCT
Forward primer *pAbp* (PCR PABC domain for cloning)	ACCGAATTCACCTCAAACATGCGC
Reverse primer *pAbp* (PCR PABC domain for cloning)	ACCCTCGAGTTATTAGTTGGCGGG
