# Supplementary material for: The PIWI protein Aubergine recruits eIF3 to activate translation in the germ plasm
Source: Cell Res. 2020 Mar 4;30(5):421–35. doi: 10.1038/s41422-020-0294-9 (PMC7196074; doi:10.1038/s41422-020-0294-9)
Supplement: Supplementary file 10 — Supplementary information, Table S4 [file 41422_2020_294_MOESM10_ESM.pdf]

Table S4 Plasmids constructed in this study

pPHW-attB
pUASp-HA- eIF3d
pUASp-HA- eIF3d^{helix11}
pAct-FLAG-Firefly-Aub
pAct-HA-Renilla-eIF3d
pAct-HA-Renilla-eIF3b
pAct-HA-Renilla-eIF3f
pAct-HA-Renilla-eIF3g
pAct-HA-Renilla-eIF3h
pAct-HA-Renilla-eIF3k
pAct-HA-Renilla-eIF4A
pAct-HA-Renilla-eIF4E
pAct-HA-Renilla-PABP
pGEX-PABP-RRM1
pGEX-PABP-RRM2
pGEX-PABP-RRM3
pGEX-PABP-RRM4
pGEX-PABP-PABC
